# Biocatalytic decarboxylative Michael addition for synthesis of 1,4-benzoxazinone derivatives

**DOI:** 10.1038/s41598-022-16291-3

**Published:** 2022-07-26

**Authors:** Hossein Bavandi, Mansour Shahedi, Zohreh Habibi, Maryam Yousefi, Jesper Brask, Mehdi Mohammadi

**Affiliations:** 1 Department of Pure Chemistry, Faculty of Chemistry, Shahid Beheshti University, G.C., Tehran, Iran; 2 Nanobiotechnology Research Center, Avicenna Research Institute, ACECR, Tehran, Iran; 3 Novozymes A/S, Krogshøjvej 36, 2880 Bagsværd, Copenhagen Denmark; 4 Bioprocess Engineering Department, Institute of Industrial and Environmental Biotechnology, National Institute of Genetic Engineering and Biotechnology (NIGEB), Tehran, Iran

**Keywords:** Green chemistry, Synthetic chemistry methodology

## Abstract

The *Candida antarctica* lipase B (Novozym 435) is found to catalyze a novel decarboxylative Michael addition in vinylogous carbamate systems for the synthesis of 1,4-benzoxazinone derivatives. The reaction goes through Michael addition, ester hydrolysis and decarboxylation. A possible mechanism is suggested, with simultaneous lipase-catalyzed Michael addition and ester hydrolysis. The present methodology offers formation of complex products through multi-step reactions in a one pot process under mild and facile reaction conditions with moderate to high yields (51–90%) and no side product formation. The reaction seems to be is a great example of enzymatic promiscuity.

## Introduction

Heterocyclic compounds containing oxygen and nitrogen are of considerable importance due to their occurrence in various natural products and their potential biological activities^1^. 1,4-benzoxazinone derivatives are examples of such heterocycles, exhibiting a wide range of biological activities such as antioxidant^2^, anti‐Alzheimer^3^, antidiabetic^4^, antimalarial^5^, antimicrobial^6^, antibacterial^7^ and anticancer^8^.

1,4-Benzoxazinones belong to a reactive class of compounds known as vinylogous carbamates^9,10^. In recent years, various efficient transformations have been developed for synthesizing these heterocyclic derivatives. Peddinti and co-workers have reported synthesis of various compounds such as pyrrolobenzoxazine^11,12^, conjugate addition reactions with *N*-substituted maleimide derivatives^13^, and regioselective 1,6-conjugate addition of 1,4-benzoxazinone to *p*-quinone methides^14^. In other works, a chiral phosphoric acid catalyzed the enantioselective addition of indole to a ketimine ester and produced new derivatives of 1,4-benzoxazinones^15,16^.

The concept of green chemistry is intrinsically linked to enzymatic catalysis, as enzymes can be obtained from renewable sources, and are capable of catalyzing various chemical reactions, being an alternative to the classical chemical catalysis^17–21^. The ability of enzymes to catalyze multiple distinctly different reactions is referred to as enzyme promiscuity^22–24^, Lipases (EC 3.1.1.3, carboxylesterase enzyme)^25,26^ have previously shown unexpected activities and have been used in organic reactions such as Aldol condensation^27^, Hantzsch reaction^28^, Cannizzaro reaction^29^, Mannich reaction^30^, Baylis–Hillman reaction^31^, Knoevenagel condensation^32^, Michael addition^33^ and Ugi reaction^34^.

As the field of biocatalysis continues to expand and play a greater role in synthetic chemistry, it is reasonable to expect that the development of innovative one-pot enzymatic processes will likewise see continued growth. The carbon–carbon (C–C) bond constructs the ‘*backbone*’ of organic molecules, and so carbon–carbon bond formation is a fundamental transformation in organic chemistry. Michael reaction in which 1,4- addition of a carbon nucleophile to an alpha/beta unsaturated carbonyl compound occurs, usually need strong acids and bases. In this study, CAL-B enzyme catalyzes the reaction under mild conditions (NO strong acids or bases) to obtain the final product. Interestingly, application of lipase catalysis in decarboxylative aldol reactions has been demonstrated to circumvent the traditional harsh conditions, such as those afforded by strong bases or metal catalysts^35^.

Decarboxylation reactions in organic chemistry are often carried out under harsh conditions such as transition metals and high temperatures. These metals are toxic and produce many by-products. It is environmentally friendly to use the Novozym 435 to synthesize 1,4-benzoxazinone derivatives because toxic metals are not used in this reaction.

**Figure 1:**
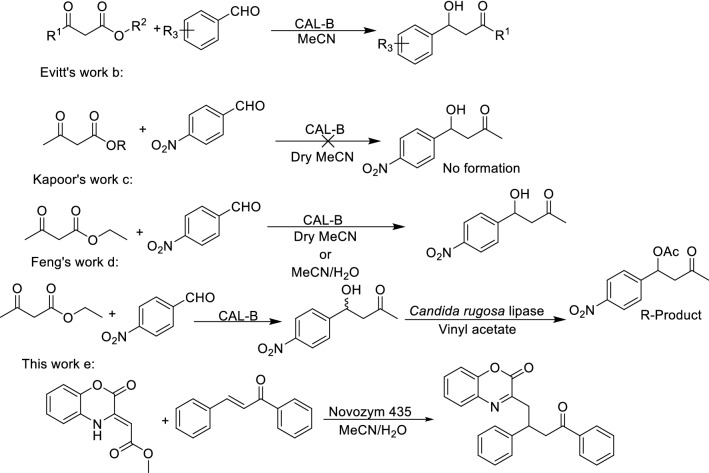
CAL-B catalyzed decarboxylative aldol and Michael reaction.

Feng et al. in 2009 reported a novel decarboxylative aldol and Knoevenagel reaction in the presence of *Candida antarctica* lipase B as a biocatalyst and acetonitrile/water as solvent (Fig. 1a)^36^. The authors investigated the catalytic effect of CAL-B by performing control experiments and found that the reaction of 4-nitrobenzaldehyde with ethyl acetoacetate in the absence of active CAL-B gave no product formation. Since experiments using acetone instead of ethyl acetoacetate also resulted in no product, it was concluded that decarboxylation was not occurring before addition. Evitt and Bornscheuer in 2011 objected to the report of Feng and coworkers (Fig. 1b), providing data suggesting the acetonitrile in Feng’s work was not adequately dry and may have contained sufficient water to promote ester hydrolysis, thereby allowing non-enzymatic aldol condensation with 4-nitrobenzaldehyde^37^. When the reaction was repeated with dry HPLC grade acetonitrile (MeCN) no aldol product and a very low formation of the Knoevenagel product were found.

However, in 2012 Kapoor et al. reinvestigated the reaction with different CAL-B formulations under presumably anhydrous conditions and found significant levels of aldol reaction^38^ (Fig. 1c), Without going in details with the mechanism, Feng et al. in 2014 reported a two-step sequential biocatalytic process for the synthesis of chiral hydroxyesters by a combination of lipase-catalyzed decarboxylative aldol reactions followed by lipase-catalyzed kinetic resolution of the secondary alcohols^39^ (Fig. 1d).

Consequently, inspired by previous works and our investigation, we presented new methods for the efficient synthesis of bioactive molecules. To the best of our knowledge, we have carried out for the first time a decarboxylation/Michael reaction between 1,4-benzoxazinone and chalcone derivatives in the presence of Novozym 435 as a biocatalyst (Fig. 1e).

## Results and discussion

Initially a handful of commercially available enzymes and proteins were investigated as biocatalysts for the decarboxylative Michael reaction (Table 1). The reaction was performed with 1,4-benzoxazinon 1 and chalcone 2 in MeCN-H_2_O (100:1). The results showed that only Novozym 435 catalyzed the reaction (entry 5). Controls with urea-denatured Novozym 435 and bovine serum albumin (BSA) protein (entry 6, 7) showed no product formation.

**Table 1:** Enzymatic screening for decarboxylative Michael reaction.

Entry	Enzyme	Yield^a^ (%)
1	*Porcine pancreas* lipase	Trace
2	Amano lipase A from *Aspergillus niger*	0
3	*Thermomyces lanuginosus* lipase	0
4	Trypsin from porcine pancreas	0
5	Novozym 435	52
6	Novozym 435 (denatured)^b^	0
7	BSA	0

Reaction conditions: 1,4 benzoxazinon (0.2 mmol), chalcone (0.2 mmol), MeCN (2 ml), H_2_O (20 µl), catalyst (10 mg), Temperature (40 °C). ^a^Isolated yield. ^b^Novozym 435 was denatured with 8 M urea for 8 h at 100 °C.

Next, reaction conditions were optimized with Novozym 435 (Table 2). The reaction was monitored by thin-layer chromatography. Since the reaction medium affects the activity of enzyme has an important role in enzymatic reactions. For instance, some organic solvents decrease activity or inactive the enzyme. In this study different solvents dimethyl sulfoxide (DMSO), dimethylformamide (DMF), tetrahydrofuran (THF), dichloromethane (DCM), MeCN were investigated (entry 1–5). Accordingly, when the reaction was carried out in DMSO or DMF, no product could be detected, and in THF and DCM yields were 10, 15% respectively. MeCN was the superior solvent, producing the Michael product **3** in the highest yield after 24 h (entry 5). The temperature optimum seems to be 40–50 °C (entry 6–8). Increasing the amount of enzyme resulted in up to 70% yields (entry 9–11); when enzyme amount is increased, more active sites are involved in the reaction. And a further improvement was obtained by increasing water concentration in the system. As the amount of water increases, the rate of hydrolysis of the ester to acid increases. Also, water clustering on the surface of enzyme plays an important role in the catalytic activity of the biocatalyst and water molecules have tended binding site at the surface of the enzyme. As more water molecules bind to the enzyme, fewer substrate molecules approach the catalytic site of CALB, and thus the active site of the enzyme becomes closed. (entry 12–14). The optimized system (entry 13) resulted in with 75% yield.

**Table 2:** Optimization of the reaction conditions.

Entry	Solvent	Temperature (°C)	Enzyme loading (mg)	Water (µl)	Time (h)	Yield^a^ (%)
1	DMSO	r.t	10	20	24	n.r
2	DMF	r.t	10	20	24	n.r
3	THF	r.t	10	20	24	10
4	DCM	r.t	10	20	24	15
5	MeCN	r.t	10	20	24	45
6	MeCN	40	10	20	24	55
7	MeCN	50	10	20	24	57
8	MeCN	60	10	20	24	43
9	MeCN	40	20	20	24	65
10	MeCN	40	30	20	24	70
11	MeCN	40	40	20	24	70
12	MeCN	40	30	30	24	72
13	MeCN	40	30	40	24	75
14	MeCN	40	30	50	24	75

Reaction conditions: 1,4-benzoxazinon (0.2 mmol), chalcone (0.2 mmol), MeCN (2 ml), H_2_O.

^a^Isolated yields.

**Figure 2:**
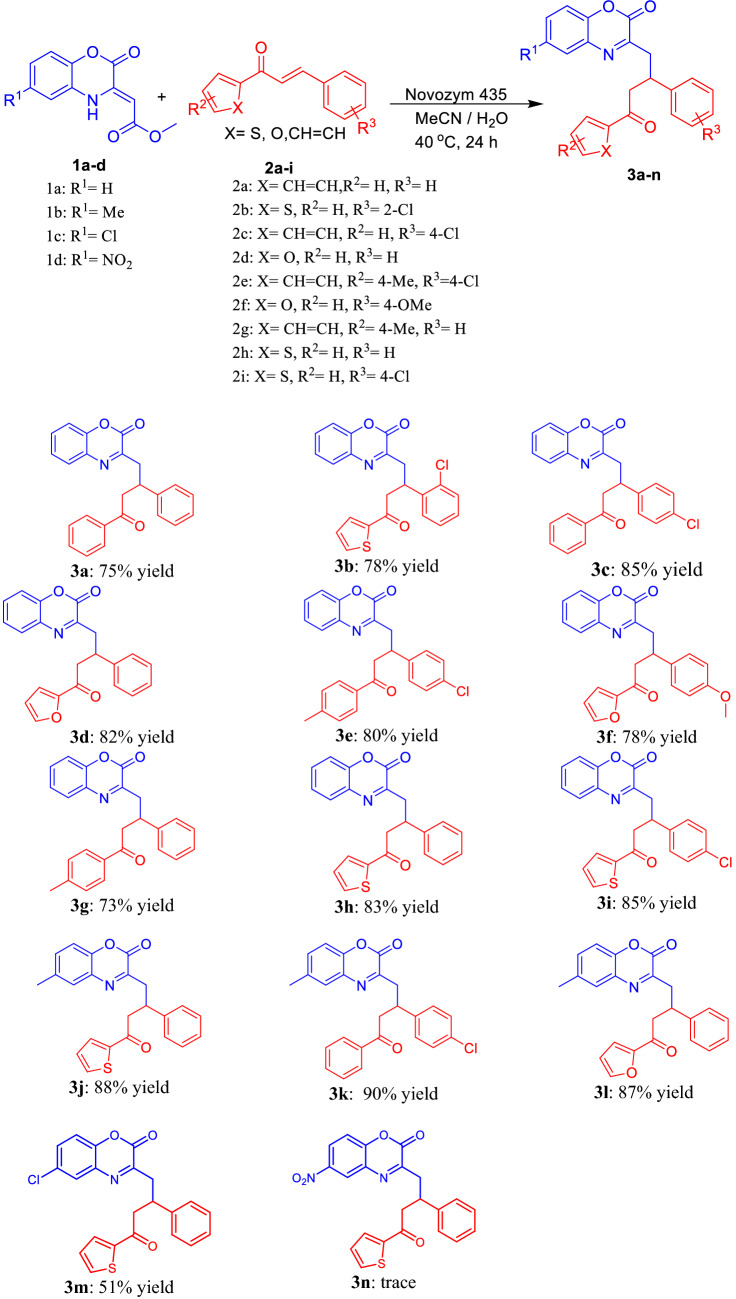
The reaction of 1,4-benoxazinone derivatives **1a–d** (0.2 mmol) with chalcone derivatives **2a–i** (0.2 mmol), MeCN (2 ml), H_2_O (40 µl), 40 °C, 24 h.

Finally, the substrate scope of the reaction was explored by using various 1,4-benzoxazinons **1a–d** and chalcones **2a–i** (Fig. 2). A variety of 1,4-benzoxazinon and chalcone derivatives bearing electron-donating and electron-withdrawing groups at the benzene ring were amenable to the reaction and produced products **3a–n** with 51–90% yields. The substitution on the aryl rings of **2a–i** has a significant effect on the yield of the products. It is evident from Fig. 2 that when R^3^ is Cl, the yield increases compared to non-substituted chalcones. This increased yield may be attributed to the fact that chlorinated chalcones can be better Michael acceptors. When R^1^ on 1,4-benzoxazinone **1a–d** is CH_3_, the yield was increased compared to non-substituted; however, with Cl and NO_2_ R^1^ substitutions yields decreased to 51% and traces, respectively. This shows electron-withdrawing groups on 1,4-benzoxazine decreased the nucleophilic capability of the Michael donor. By having withdrawing groups on the chalcon benzene ring (R^3^), it is suitable for nucleophilic attack due to its lower electron current. Furthermore, when the withdrawing groups are attached to the benzoxazinone ring (R^1^), the electron density and nucleophilicity of the compound decrease.

To investigate the mechanism of the reaction, several control experiments were performed.

**Figure 3:**
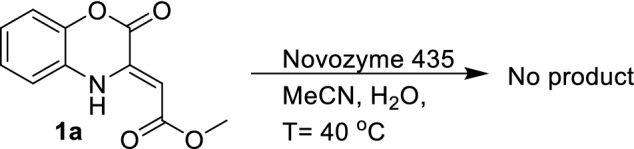
Control experiment for decarboxylative Michael reactions.

At first the reaction was carried out with 1,4 benzoxazinone derivative (**1a**) in the absence of chalcone, in presence of MeCN and water as solvent (Fig. 3). Under these conditions, no ester hydrolysis or decarboxylation could be detected within 48 h reaction.

Secondly, the reaction was performed with dried acetonitrile and no added water. This reaction resulted in decarboxylative Michael addition product in 24 h, indicating hydrolysis prior to Michael addition is not critical for the reaction.

**Figure 4:**
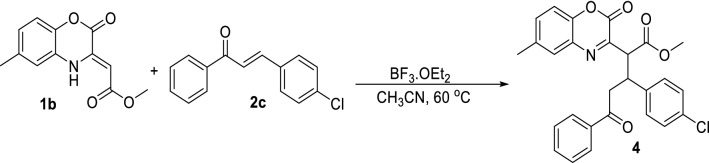
Control experiment for BF_3_ catalyzed Michael addition.

In a third control reaction, we performed the Michael addition of chalcone (**2c**) and 1,4-benzoxazinone derivative (**1b**) through chemical catalysis by BF_3_.OEt_2_ (Fig. 4). Subsequently the product (**4**) was incubated with Novozym 435, but after even 48 h no product was formed (according to TLC and HPLC comparison with our previous product).

In retrospect, it is not surprising that ester hydrolysis was not observed in control experiments 1 and 3, since both substrates contain bulky substitutions on the carboxylic acid side of the ester, something which most lipases, including CAL-B, struggle with^40^. In contrast to the broad spectrum of alcohol moieties accepted as substrates, only a limited spectrum of acids is accepted by CAL-B. For instance, acyl moieties with sterically demanding α- and β-substitutions yield significantly reduced specific activities^41,42^.

**Figure 5:**
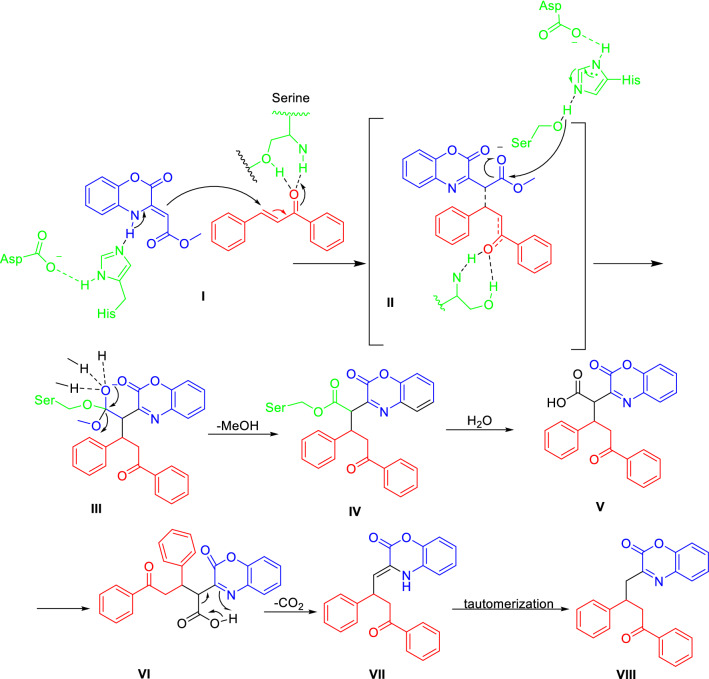
Suggested mechanism of decarboxylative Michael reaction.

A proposal mechanism based on previous works^30,43,44^ and control experiments for the formation of the Michael adduct is presented in Fig. 5. The lipase catalyzed reaction involves the amino acids known as the catalytic triad composed of serine (Ser), histidine (His), and aspartate (Asp) in the enzyme active site. First, the carbonyl group in chalcone is activated by hydrogen bonding to serine, then 1,4-benzoxazinone is deprotonated by the His-Asp system, setting it up for nucleophilic attack of the vinylogous carbamate on the activated chalcone Michael acceptor (**I**). By considering the control experiments, it seems that the lipase-catalyzed ester hydrolysis occurs simultaneously in an intermediate (**II**) involving both chalcone and 1,4-benzoxazinone. In this way, the ester is first activated with the lipase, likely because of the charge delocalization facilitating nucleophilic attack of the serine residue. Subsequently, carbon dioxide is released from (**VI**) and Finally, the enamine (**VII**) is converted to the final product (**VIII**).

## Conclusion

In conclusion, we have successfully developed an efficient biocatalytic methodology for the synthesis of 1,4-benzoxazinone derivatives. The reaction seems to proceed via lipase catalyzed nucleophilic attack of 1,4-benzoxazinones to chalcone Michael acceptors and methyl ester hydrolysis followed by decarboxylation in the presence of Novozym 435. This reaction can be carried out under mild conditions with moderate to excellent yields of 1,4-benzoxazinone derivatives. This novel approach extends the already wide application of Novozym 435 in organic chemistry and provides an effective and environmentally friendly synthetic route for synthesis of 1,4-benzoxazinone derivatives.

## Experimental

### General information and methods

Commercial chemicals were used without further purification. Immobilized *Candida antarctica* lipase B (Novozym 435) was kindly donated by Novozymes Denmark. Porcine pancreas lipase (EC 3.1.1.3), *Thermomyces lanuginosus* lipase (EC 3.1.1.3), Amano lipase from *Aspergillus niger* (EC 3.1.1.3), and trypsin from porcine pancreas (EC 3.4.21.4) were purchased from Sigma-Aldrich. Analytical TLC (thin-layer chromatography) was performed on Merck pre-coated [silica gel 60 F254 20 × 20 cm)] plates. Melting points were determined with a melting point Thermo Scientific 9100 apparatus and are uncorrected. IR spectra were taken with a Bomem FT-IR MB spectrometer. NMR spectra were recorded in CDCl_3_ with 300 MHz Bruker DRX Avance spectrometers. Mass spectra were recorded with an Agilent Technologies (HP) 5975C mass spectrometer by electron ionization (EI) (20–70 eV).

### Synthesis of 1,4-benzoxazinone derivatives 1a–d

1,4-Benzoxazinones were prepared according to Peddinti's work^44^, Hence aminophenols (2-Amino-4-methyl phenol, 2-amino-4-chlorophenol, 2-amino-4-nitrophenol) (5 mmol) and dimethyl acetylenedicarboxylate (5 mmol) were mixed in a glass beaker for 2–5 min with the help of a spatula to form a homogeneous paste. The reaction was completed within several minutes and afforded a solid product, which was washed with a few drops of methanol.

### Synthesis of chalcone derivatives 2a–i

To a solution of ketone (acetophenone, 4-methylacetophenone, 2-acetylthiophene, 2-acetylfuran) (10 mmol) and aldehyde (benzaldehyde, 4-chlorobenzaldehyde, 2-chlorobenzaldehyde, and 4-methoxybenzaldehyde) (10 mmol) in 20 ml of methanol on an ice bath, 8 ml 10% NaOH was added dropwise over 10 min. After that, the reaction was stirred overnight at room temperature. The precipitate was separated by filtration and washed three times with a mixture of 1:1 ethanol:water. If needed, the crude product was recrystallized from hot ethanol.

### General procedure for the synthesis of products 3a–n

To a mixture of 1,4-benzoxazinone **1a–d** (0.2 mmol), chalcones **2a–i** (0.2 mmol) in 2 ml of MeCN solvent, 40 µl H_2_O, Novozym 435 (30 mg) was added and the mixture was stirred at 40 °C (120 rpm) for 24 h. The reaction was monitored by TLC. The Novozym 435 was filtered and the solution concentrated under reduced pressure and the crude product was purified by thin layer chromatography on silica gel plates using *n*-hexane/ethylacetate (5:1) to yield pure compound **3a-n**.

### Characterization of products 3a–m

#### 3-(4-oxo-2,4-diphenylbutyl)-2H-benzo[b][1,4]oxazin-2-one (3a)

Solid (light yellow), Isolated yield = 0.055 g [75%], Melting point: 124–126 °C; IR (ν_max_/cm^−1^): 3066, 1737, 1668; ^1^H NMR (300 MHz, Chloroform-*d*) δ 7.89–7.80 (m, 2H), 7.64–7.45 (m, 2H), 7.50–7.38 (m, 3H), 7.44–7.15 (m, 7H), 4.24 (m, 1H), 3.56 (dd, *J* = 17.1, 7.6 Hz, 1H), 3.49–3.34 (m, 2H), 3.23 (dd, *J* = 15.3, 8.2 Hz, 1H). ^13^C NMR (75 MHz, Chloroform-*d*) δ 198.6, 156.3, 153.0, 146.3, 144.0, 136.9, 133.0, 130.9, 133.5, 128.7, 128.6, 128.5, 127.9, 127.5, 126.7, 125.2, 116.3, 44.8, 40.7, 38.0; MS (EI, 70 eV): *m/z* = 369 [M^+^]. Anal. Calcd for C_24_H_19_NO_3_: C = 78.03, H = 5.18, N = 3.79; Found: C = 77.62, H = 5.21, N = 3.48.

#### 3-(2-(2-Chlorophenyl)-4-oxo-4-(thiophen-2-yl)butyl)-2H-benzo[b][1,4]oxazin-2-one(3b)

Solid (light yellow), Isolated yield = 0.063 g [78%], Melting point: 114–116 °C; IR (ν_max_/cm^−1^): 3095, 1735, 1650; ^1^H NMR (300 MHz, Chloroform-*d*) δ 7.84–7.69 (m, 1H), 7.59 (t, *J* = 6.4 Hz, 2H), 7.41 (td, *J* = 17.2, 7.8 Hz, 3H), 7.26 (d, *J* = 10.6 Hz, 4H), 7.20–7.01 (m, 2H), 4.81–4.58 (m, 1H), 3.48 (dd, *J* = 16.6, 7.7 Hz, 1H), 3.39 (q, *J* = 9.4, 7.5 Hz, 3H). ^13^C NMR (75 MHz, CDCl_3_) δ 191.0, 155.5, 152.9, 146.3, 144.1, 140.7, 133.9, 133.8, 131.9, 130.9, 130.6, 129.9, 128.8, 128.0, 127.9, 127.1, 125.2, 116.3, 44.2, 38.6, 34.9; MS (EI, 70 eV): *m/z* = 409 [M^+^]. Anal. Calcd for C_22_H_16_ClNO_3_S: C = 64.47, H = 3.93, N = 3.42; Found: C = 64.15, H = 3.77, N = 2.99.

#### 3-(2-(4-Chlorophenyl)-4-oxo-4-phenylbutyl)-2H benzo[b][1,4]oxazin-2-one (3c)

Solid (light brown), Isolated yield = 0.068 g [85%], Melting point: 138–140 °C; IR (ν_max_/cm^−1^): 3056, 1737, 1679; ^1^H NMR (300 MHz, Chloroform-*d*) δ 7.86 (d, *J* = 7.8 Hz, 2H), 7.61 (d, *J* = 7.9 Hz, 1H), 7.59–7.39 (m, 4H), 7.38 (dd, *J* = 21.1, 13.2 Hz, 6H), 4.23 (m, 1H), 3.53 (dd, *J* = 17.3, 7.2 Hz, 1H), 3.38 (dd, *J* = 16.2, 6.8 Hz, 2H), 3.22 (dd, *J* = 15.6, 7.9 Hz, 1H). ^13^C NMR (75 MHz, CDCl_3_) δ 198.2, 155.8, 153.0, 146.3, 142.4, 136.7, 133.2, 132.3, 130.9, 130.7, 129.0, 128.7, 128.5, 127.9, 125.3, 116.3, 44.7, 40.3, 37.3; MS (EI, 70 eV): *m/z* = 403 [M^+^]. Anal. Calcd for C_24_H_18_ClNO_3_: C = 71.38, H = 4.49, N = 3.47; Found: C = 71.53, H = 4.79, N = 3.51.

#### (4-(Furan-2-yl)-4-oxo-2-phenylbutyl)-2H-benzo[b][1,4]oxazin-2-one (3d)

Solid (light yellow), Isolated yield = 0.059 g [82%], Melting point: 127–129 °C. IR (ν_max_/cm^−1^): 3070, 1735, 1660; ^1^H NMR (300 MHz, Chloroform-*d*) δ 7.63 (d, *J* = 7.7 Hz, 1H), 7.54 (s, 1H), 7.45 (d, *J* = 7.8 Hz, 1H), 7.43 – 7.25 (m, 5H), 7.21 (q, *J* = 9.0, 7.5 Hz, 1H), 7.09 (t, *J* = 3.0 Hz, 1H), 6.79 (s, 1H), 6.49 (s, 1H), 4.19 (m, 1H), 3.44–3.16 (m, 4H). ^13^C NMR (75 MHz, CDCl_3_) δ 187.6, 156.0, 152.7, 146.2, 143.6, 130.9, 130.6, 128.7, 128.6, 127.5, 126.7, 125.2, 117.0, 116.3, 112.2, 44.6, 40.4, 38.0; MS (EI, 70 eV): *m/z* = 359 [M^+^]. Anal. Calcd for C_22_H_17_NO_4_: C = 73.53, H = 4.77, N = 3.90; Found: C = 73.33, H = 4.85, N = 3.88.

#### 3-(2-(4-Chlorophenyl)-4-oxo-4-(p-tolyl)butyl)-2H-benzo[b][1,4]oxazin-2-one (3e)

Solid (light yellow), Isolated yield = 0.067 g [80%], Melting point: 157–160 °C; IR (ν_max_/cm^−1^): 3075, 1735, 1675; ^1^H NMR (300 MHz, Chloroform-*d*) δ 7.76 (d, *J* = 8.0 Hz, 2H), 7.62 (d, *J* = 8.0 Hz, 1H), 7.47 (t, *J* = 7.9 Hz, 1H), 7.27 (td, *J* = 25.4, 7.9 Hz, 8H), 4.22 (m, 1H), 3.50 (dd, *J* = 17.1, 7.1 Hz, 1H), 3.36 (d, *J* = 17.5, 7.2 Hz, 2H), 3.21 (dd, *J* = 15.3, 7.8 Hz, 1H), 2.41 (s, 3H). ^13^C NMR (75 MHz, CDCl_3_) δ 197.8, 155.8, 153.0, 146.3, 144.0, 142.4, 134.3, 132.3, 130.9, 130.6, 129.2, 129.0, 128.7, 128.7, 128.0, 125.3, 116.3, 44.5, 40.3, 37.4, 21.6; MS (EI, 70 eV): *m/z* = 417 [M^+^]. Anal. Calcd for C_25_H_20_ClNO_3_: C = 71.86, H = 4.82, N = 3.35; Found: C = 71.94, H = 4.73, N = 3.10.

#### 3-(4-(Furan-2-yl)-2-(4-methoxyphenyl)-4-oxobutyl)-2H-benzo[b][1,4]oxazin-2-one (3f)

Solid (light yellow), Isolated yield = 0.060 g [78%], Melting point: 118–120 °C. IR (ν_max_/cm^−1^): 3019, 1732, 1604; ^1^H NMR (300 MHz, Chloroform-*d*) δ 7.63 (d, *J* = 8.0 Hz, 1H), 7.54 (d, *J* = 9.4 Hz, 1H), 7.45 (t, *J* = 7.9 Hz, 1H), 7.29 (td, *J* = 12.1, 7.6 Hz, 4H), 7.10 (dd, *J* = 10.3, 3.7 Hz, 1H), 6.82 (d, *J* = 8.5 Hz, 2H), 6.58–6.35 (m, 1H), 4.15 (m, 1H), 3.78 (s, 3H), 3.41–3.15 (m,4H). ^13^C NMR (75 MHz, CDCl_3_) δ 187.7, 158.2, 156.1, 152.9, 152.7, 146.3, 135.5, 131.0, 130.6, 128.7, 128.5, 125.2, 117.0, 116.3, 113.9, 112.2, 55.1, 44.8, 40.6, 37.3; MS (EI, 70 eV): *m/z* = 389 [M^+^]. Anal. Calcd for C_23_H_19_NO_5_: C = 70.94, H = 4.92, N = 3.60; Found: C = 70.90, H = 4.79, N = 3.41.

#### 3-(4-Oxo-2-phenyl-4-(p-tolyl) butyl)-2H-benzo[b][1,4]oxazin-2-one (3g)

Solid (light yellow), Isolated yield = 0.056 g [73%], Melting point: 142–146 °C; IR (ν_max_/cm^−1^): 3039, 1733, 1670; ^1^H NMR (300 MHz, Chloroform-*d*) δ 7.75 (d, *J* = 7.8 Hz, 2H), 7.60 (d, *J* = 7.8 Hz, 2H), 7.53–7.36 (m, 3H), 7.35–7.24 (m, 3H), 7.20 (d, *J* = 7.7 Hz, 3H), 4.23 (m, 1H), 3.53 (dd, *J* = 17.1, 7.7 Hz, 1H), 3.39 (dd, *J* = 14.3, 12.1, 8.5 Hz, 2H), 3.22 (dd, *J* = 15.3, 8.1 Hz, 1H), 2.40 (s, 3H). ^13^C NMR (75 MHz, CDCl_3_) δ 198.2, 156.3, 153.0, 146.3, 144.0, 143.8, 134.4, 131.0, 130.5, 129.1, 128.7, 128.6, 128.1, 127.5, 126.7, 125.2, 116.3, 44.7, 40.7, 38.1, 21.6; MS (EI, 70 eV): *m/z* = 383 [M^+^]. Anal. Calcd for C_25_H_21_NO_3_: C = 78.31, H = 5.52, N = 3.65; Found: C = 78.27, H = 5.41, N = 3.42.

#### 3-(4-Oxo-2-phenyl-4-(thiophen-2-yl)butyl)-2H-benzo[b][1,4]oxazin-2-one (3h)

Solid (light yellow), Isolated yield = 0.061 g [83%], Melting point: 157–160 °C; IR (ν_max_/cm^−1^): 3054, 1729, 1660; ^1^H NMR (300 MHz, Chloroform-*d*) δ 7.73–7.54 (m, 3H), 7.51–7.15 (m, 8H), 7.10 (t, *J* = 4.4 Hz, 1H), 4.23 (m, 1H), 3.43 (dd, *J* = 17.3, 16.9, 6.9 Hz, 2H), 3.25 (dd, *J* = 15.4, 7.7 Hz, 2H). ^13^C NMR (75 MHz, CDCl_3_) δ 191.4, 156.0, 146.3, 144.3, 143.6, 133.7, 131.8, 130.9, 130.5, 128.7, 128.6, 128.0, 127.5, 126.8, 125.2, 116.3, 45.5, 40.4, 38.3; MS (EI, 70 eV): *m/z* = 375 [M^+^]. Anal. Calcd for C_22_H_17_NO_3_S: C = 70.38, H = 4.56, N = 3.73; Found: C = 70.21, H = 4.51, N = 3.69.

#### 3-(2-(4-Chlorophenyl)-4-oxo-4-(thiophen-2-yl)butyl)-2H-benzo[b][1,4]oxazin-2-one (3i)

Solid (light yellow), Isolated yield = 0.069 g [85%], Melting point: 139–142 °C; IR (ν_max_/cm^−1^): 3048, 1725, 1658; ^1^H NMR (300 MHz, Chloroform-*d*) δ 7.65 (s, 3H), 7.73–7.56 (m, 2H), 7.31 (q, *J* = 16.0, 12.0 Hz, 5H), 7.19–7.06 (m, 1H), 4.30–4.15 (m, 1H), 3.54 (m, 1H), 3.33 (m, 3H). ^13^C NMR (75 MHz, CDCl_3_) δ 190.9, 155.6, 152.9, 146.3, 144.1, 142.04, 133.9, 132.4, 131.9, 130.9, 130.7, 128.9, 128.7, 128.0, 125.3, 116.3, 45.3, 40.1, 37.6; MS (EI, 70 eV): *m/z* = 409 [M^+^]. Anal. Calcd for C_22_H_16_ClNO_3_S: C = 64.47, H = 3.93, N = 3.42; Found: C = 64.57, H = 3.68, N = 3.47.

#### 6-Methyl-3-(4-oxo-2-phenyl-4-(thiophen-2-yl)butyl)-2H-benzo[b][1,4]oxazin-2-one (3j)

Solid (light brown), Isolated yield = 0.068 g [88%], Melting point: 131–135 °C; IR (ν_max_/cm^−1^): 3050, 1745, 1658; ^1^H NMR (300 MHz, Chloroform-*d*) δ 7.70 (d, *J* = 3.8 Hz, 1H), 7.59 (t, *J* = 4.1 Hz, 1H), 7.40 (d, *J* = 6.4 Hz, 3H), 7.37–7.18 (m, 4H), 7.12 (d, *J* = 12.8, 3.6 Hz, 2H), 4.23 (m, 1H), 3.36 (m, 4H), 2.40 (s, 3H). ^13^C NMR (75 MHz, CDCl_3_) δ 191.3, 155.8, 153.2, 144.3, 144.2, 143.7, 135.1, 133.6, 131.8, 131.4, 130.7, 128.6, 127.9, 127.5, 126.7, 115.8, 45.5, 40.3, 38.3, 20.7; MS (EI, 70 eV): *m/z* = 389 [M^+^]. Anal. Calcd for C_23_H_19_NO_3_S: C = 70.93, H = 4.92, N = 3.60; Found: C = 70.89, H = 4.96, N = 3.14.

#### 3-(2-(4-Chlorophenyl)-4-oxo-4-phenylbutyl)-6-methyl-2H-benzo[b][1,4]oxazin-2-one (3k)

Solid (light brown), Isolated yield = 0.075 g [90%], Melting point: 143–145 °C; IR (ν_max_/cm^−1^): 3058, 1724, 1679; ^1^H NMR (300 MHz, Chloroform-*d*) δ 7.90–7.81 (m, 2H), 7.55 (t, *J* = 7.0 Hz, 1H), 7.49–7.35 (m, 3H), 7.29 (q, *J* = 8.7 Hz, 5H), 7.15 (d, *J* = 8.3 Hz, 1H), 4.21 (m, *J* = 7.0 Hz, 1H), 3.52 (dd, *J* = 17.3, 7.0 Hz, 1H), 3.44–3.29 (m, 2H), 3.20 (dd, *J* = 15.6, 7.9 Hz, 1H), 2.41 (s, *J* = 3.8 Hz, 3H). ^13^C NMR (75 MHz, CDCl_3_) δ 198.1, 155.6, 144.2, 142.4, 136.7, 135.2, 133.1, 132.3, 131.6, 130.6, 128.9, 128.7, 128.5, 127.9, 115.9, 44.7, 40.3, 37.4, 20.7; MS (EI, 70 eV): *m/z* = 417 [M^+^]. Anal. Calcd for C_25_H_20_ClNO_3_: C = 71.86, H = 4.82, N = 3.35; Found: C = 71.94, H = 4.84, N = 3.30.

#### 3-(4-(Furan-2-yl)-4-oxo-2-phenylbutyl)-6-methyl-2H-benzo[b][1,4]oxazin-2-one (3l)

Solid (light brown), Isolated yield = 0.065 g [87%], Melting point: 136–138 °C; IR (ν_max_/cm^−1^): 3102, 1741, 1670; ^1^H NMR (300 MHz, Chloroform-*d*) δ 7.53 (s, 1H), 7.47–7.34 (m, 3H), 7.28 (q, *J* = 8.5 Hz, 3H), 7.17–7.06 (m, 3H), 6.49 (d, *J* = 3.6 Hz, 1H), 4.19 (m,1H), 3.31 (m, 4H), 2.42 (s, 3H). ^13^C NMR (75 MHz, CDCl_3_) δ 187.6, 155.8, 153.2, 152.7, 146.2, 144.2, 143.6, 135.1, 131.4, 130.7, 128.6, 128.5, 127.5, 126.7, 117.0, 115.8, 112.1, 44.6, 40.3, 38.0, 20.7; MS (EI, 70 eV): *m/z* = 373 [M^+^]. Anal. Calcd for C_23_H_19_NO_4_: C = 73.98, H = 5.13, N = 3.75; Found: C = 73.87, H = 5.24, N = 3.71.

#### 6-Chloro-3-(4-oxo-2-phenyl-4-(thiophen-2-yl)butyl)-2H-benzo[b][1,4]oxazin-2-one (3m)

Solid (brown), Isolated yield = 0.041 g [51%], Melting point: 137–140; IR (ν_max_/cm^−1^): 3116, 1743, 1639; ^1^H NMR (300 MHz, Chloroform-*d*) δ 7.68 (d, *J* = 3.8 Hz, 1H), 7.56 (dd, *J* = 22.9, 3.7 Hz, 2H), 7.45–7.26 (m, 5H), 7.22 (t, *J* = 8.3 Hz, 2H), 7.11 (t, *J* = 4.4 Hz, 1H), 4.20 (m, 1H), 3.46 (m, 4H). ^13^C NMR (75 MHz, CDCl_3_) δ 191.4, 157.5, 152.4, 144.9, 144.2, 143.5, 133.9, 131.9, 131.4, 130.4, 130.2, 128.7, 128.1, 128.0, 127.4, 126.9, 117.4, 45.5, 40.6, 38.2; MS (EI, 70 eV): *m/z* = 409 [M^+^]. Anal. Calcd for C_22_H_16_ClNO_3_S: C = 64.47, H = 3.93, N = 3.42; Found: C = 64.52, H = 3.91, N = 3.89.

## Supplementary Information

Supplementary Information.

## Data Availability

All data generated or analysed during this study are included in this published article and its supplementary information files.
