# Supplementary material for: Biocatalytic decarboxylative Michael addition for synthesis of 1,4-benzoxazinone derivatives
Source: Sci Rep. 2022 Jul 26;12:12713. doi: 10.1038/s41598-022-16291-3 (PMC9325775; doi:10.1038/s41598-022-16291-3)

**Supporting Information**

**Decarboxylative Michael addition by Novozym 435: Synthesis of 1,4-benzoxazinone derivatives**

Hossein Bavandi^a^, Mansour Shahedi ^a^, Zohreh Habibi ^a*^, Maryam Yousefi ^b**^, Jesper Brask ^c^ ,Mehdi Mohammadi ^d^

^a^ Department of Pure Chemistry, Faculty of Chemistry, Shahid Beheshti University, G.C., Tehran, Iran

^b^ Nanobiotechnology Research Center, Avicenna Research Institute, ACECR, Tehran, Iran

^c^ Novozymes A/S, Krogshøjvej 36, 2880 Bagsværd, Copenhagen, Denmark

^d^ Bioprocess Engineering Department, Institute of Industrial and Environmental Biotechnology, National Institute of Genetic Engineering and Biotechnology (NIGEB), Tehran, Iran

^*^ Corresponding author. Tel.:+982 129 903 110; fax: +982 122 431 663.E-mail addresses: Z_habibi@sbu.ac.ir (Z. Habibi)

^**^ Corresponding author. Tel.:+982 122 432 020; fax: +982 122 432 021. E-mail addresses: M.yousefi@ari.ir (M. Yousefi)

**Table of Contents**

**Control reactions**……………………..……………………………………..…S3

**^1^H, ^13^C NMR, Mass and FT-IR Spectra**………………………..……………....S6

**Control reactions**

**Hydrolysis of 1,4-benzoxazinone derivative (1a)**

To 1,4-benzoxazinone **1a** (0.1 mmol) in (MeCN 1 mL, water 20-60 µL) solvent, Novozym 435 (15 mg) was added and the mixture was stirred at 40 °C (120 rpm) for 48 h. After this time no decarboxylation was observed by TLC or HPLC analysis.

**Control reaction in dry acetonitrile**

Acetonitrile HPLC grade was dried by distillation over calcium hydride. To 1,4-benzoxazinone **1a** (0.1 mmol), chalcones **2a** (0.2 mmol) in MeCN (1 mL), Novozym 435 (15 mg) was added and the mixture was stirred at 40 °C (120 rpm) for 24 h. Then Novozym 435 was filtered and the solution concentrated under reduced pressure and the crude product was purified by thin layer chromatography on silica gel plates using *n*-hexane/ethylacetate (5:1) to yield (30%) pure compound **3a**.

**Synthesis of methyl 3-(4-chlorophenyl)-2-(6-methyl-2-oxo-2H-benzo[b][1,4]oxazin-3-yl)-5-oxo-5-phenylpentanoate (compound 4)**

Chalcone **2c** (0.2 mmol) was added to a stirred solution of 1,4-benzoxazinone derivative **1b** (0.2 mmol) in MeCN (4 mL) followed by the addition of BF_3_·OEt_2_ (0.4 mmol) at 60 °C. The mixture was stirred for 4 h, before the solvent was evaporated. The mixture was extracted with ethyl acetate, dried with anhydrous Na_2_SO_4_, and concentrated under reduced pressure. Then the crude product was purified by thin layer chromatography on silica gel plates using *n*-hexane/ethylacetate (6:1) to yield pure compound (**4**).

(**4**)

**Figure Legends:**

Figure 1S. Compound **3a** ^1^H NMR

Figure 2S. Compound **3a** ^13^C NMR

Figure 3S. Compound **3a** MS (m/z) 369 [M^+^]

Figure 4S. Compound **3a** FT-IR

Figure 5S. Compound **3b** ^1^H NMR

Figure 6S. Compound **3b** ^13^C NMR

Figure 7S. Compound **3b** MS (m/z) 409 [M^+^]

Figure 8S. Compound **3b** FT-IR

Figure 9S. Compound **3c** ^1^H NMR

Figure 10S. Compound **3c** ^13^C NMR

Figure 11S. Compound **3c** MS (m/z) 403 [M^+^]

Figure 12S. Compound **3c** FT-IR

Figure 13S. Compound **3d** ^1^H NMR

Figure 14S. Compound **3d** ^13^C NMR

Figure 15S. Compound **3d** MS (m/z) 359 [M^+^]

Figure 16S. Compound **3e** ^1^H NMR

Figure 17S. Compound **3e** ^13^C NMR

Figure 18S. Compound **3e** MS (m/z) 417 [M^+^]

Figure 19S. Compound **3f** ^1^H NMR

Figure 20S. Compound **3f** ^13^C NMR

Figure 21S.Compound **3f** MS (m/z) 389 [M^+^]

Figure 22S.Compound **3g** ^1^H NMR

Figure 23S.Compound **3g** ^13^C NMR

Figure 24S. Compound **3g** MS (m/z) 383 [M^+^]

Figure 25S. Compound **3h** ^1^H NMR

Figure 26S. Compound **3h** ^13^C NMR

Figure 27S. Compound **3h** MS (m/z) 375 [M^+^]

Figure 28S. Compound **3i** ^1^H NMR

Figure 29S. Compound **3i** ^13^C NMR

Figure 30S. Compound **3i** MS (m/z) 409 [M^+^]

Figure 31S. Compound **3j** ^1^H NMR

Figure 32S. Compound **3j** ^13^C NMR

Figure 33S. Compound **3j** MS (m/z) 389 [M^+^]

Figure 34S. Compound **3k** ^1^H NMR

Figure 35S. Compound **3k** ^13^C NMR

Figure 36S. Compound **3k** MS (m/z) 417 [M^+^]

Figure 37S. Compound **3l** ^1^H NMR

Figure 38S. Compound **3l** ^13^C NMR

Figure 39S. Compound **3l** MS (m/z) 373 [M^+^]

Figure 40S. Compound **3m** ^1^H NMR

Figure 41S. Compound **3m** ^13^C NMR

Figure 42S. Compound **3m** MS (m/z) 409 [M^+^]

**^1^H, ^13^C NMR, Mass and FT-IR Spectra**

**Figure 1S. Compound 3a ^1^H NMR**

**
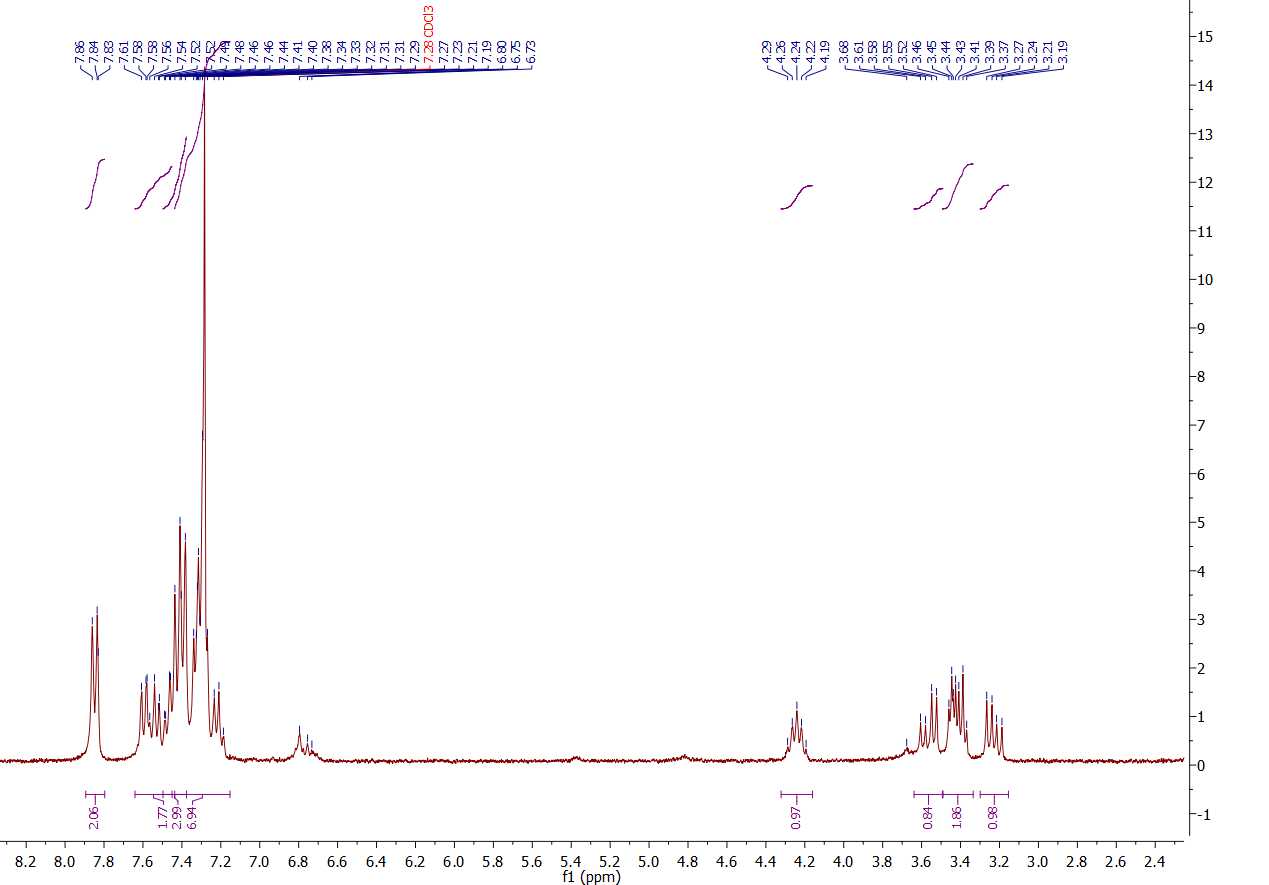
**

**Figure 2S. Compound 3a ^13^C NMR**

**
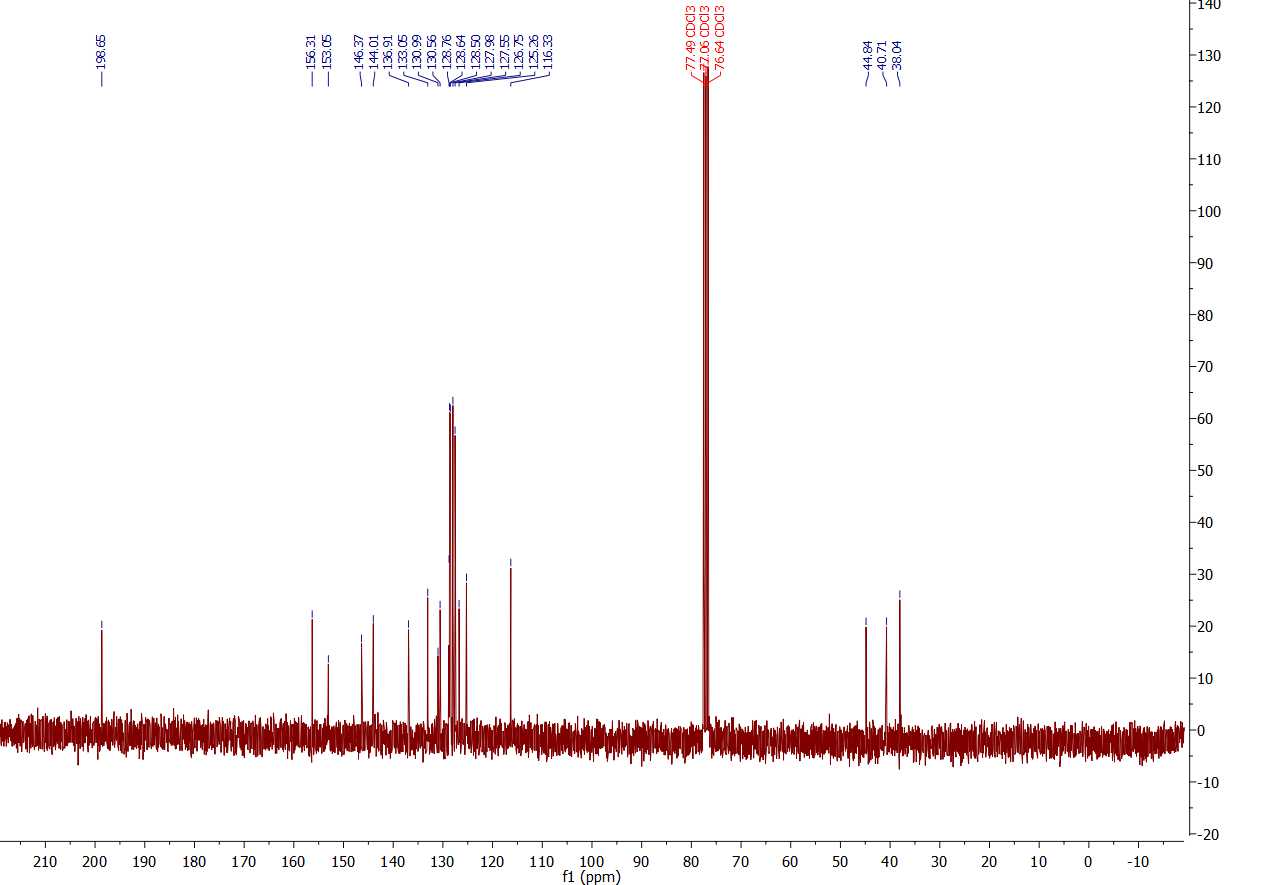
**

**Figure 3S. Compound 3a MS (m/z) 369 [M^+^]**


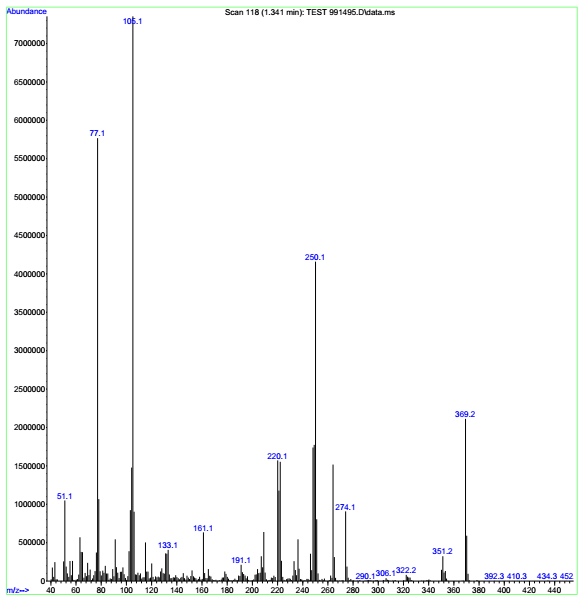

**Figure 4S. Compound 3a** **FT-IR**


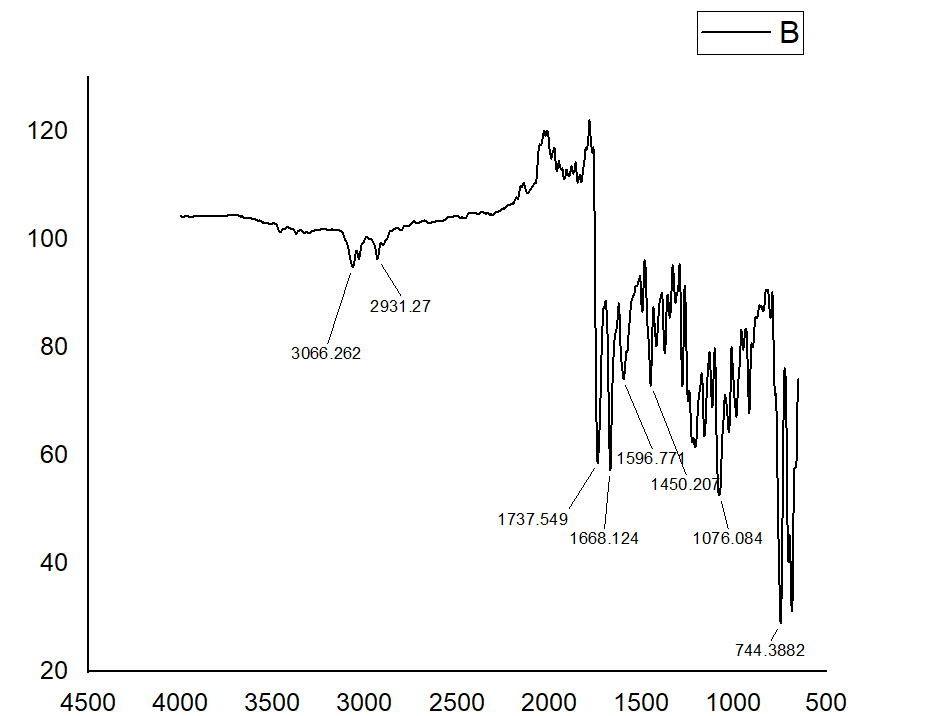

**Figure 5S. Compound 3b ^1^H NMR**

**
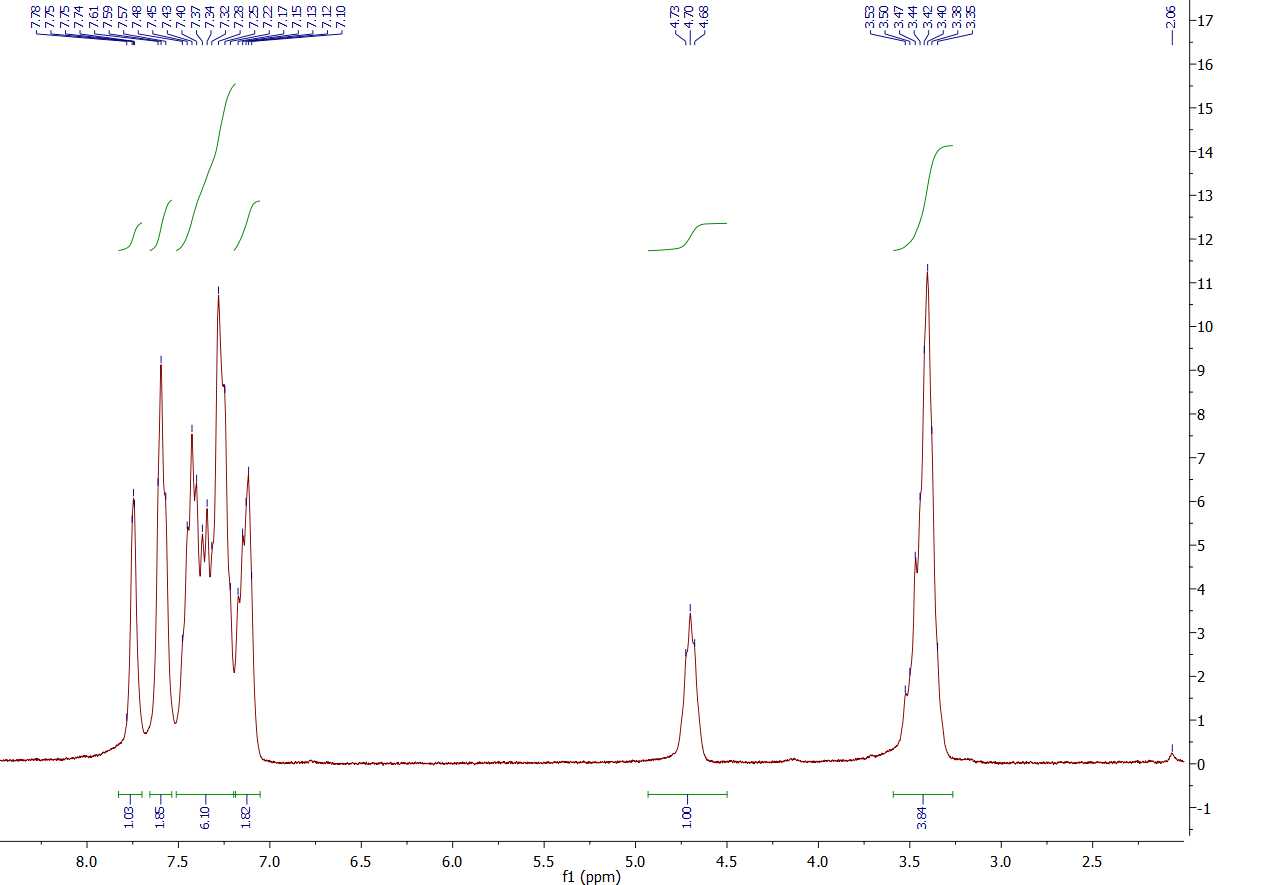
**

**Figure 6S. Compound 3b ^13^C NMR**


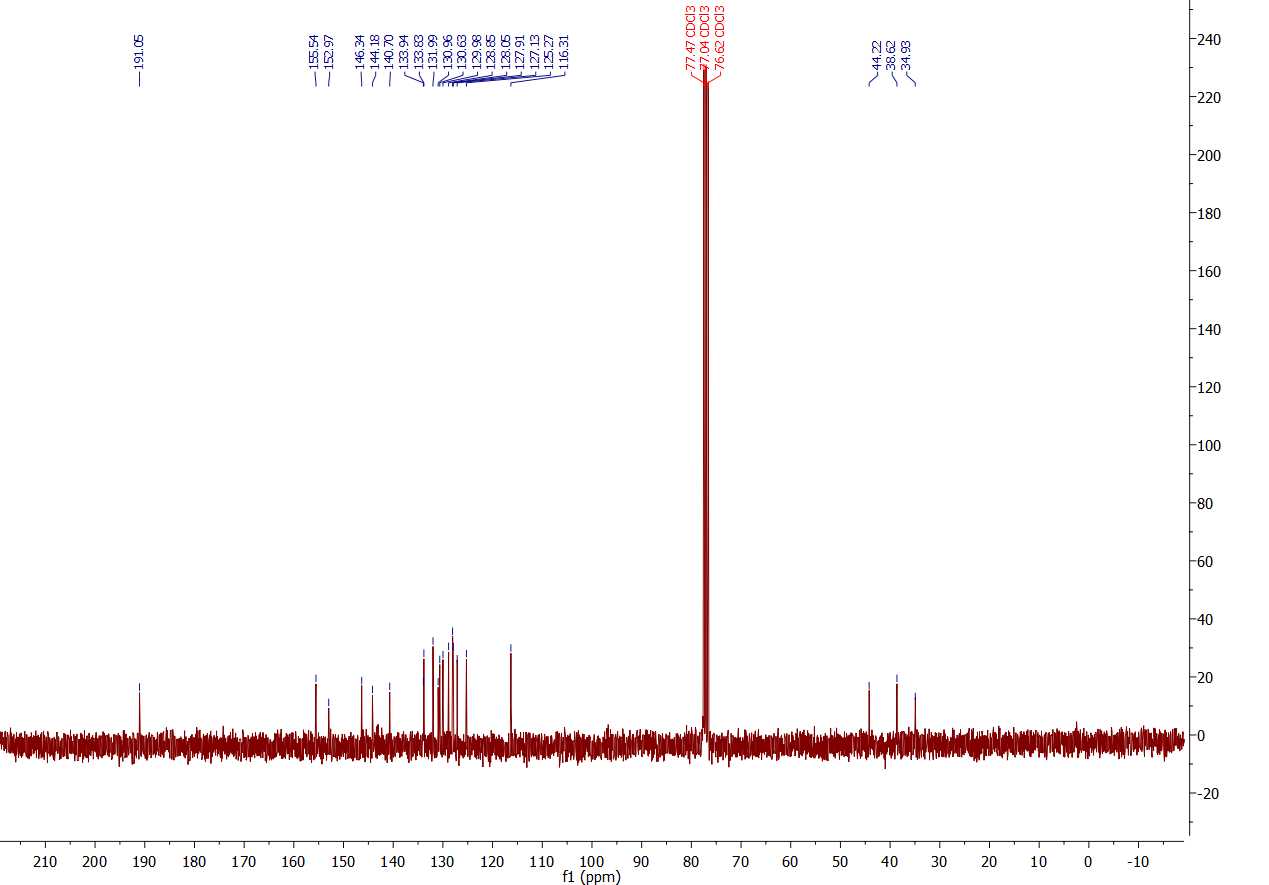

**Figure 7S. Compound 3b MS (m/z) 409 [M^+^]**


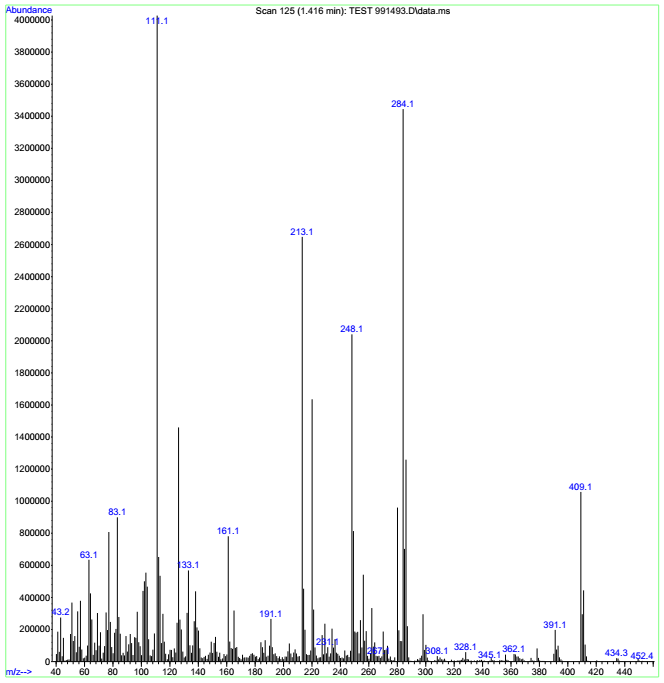

**Figure 8S. Compound 3b** **FT-IR**


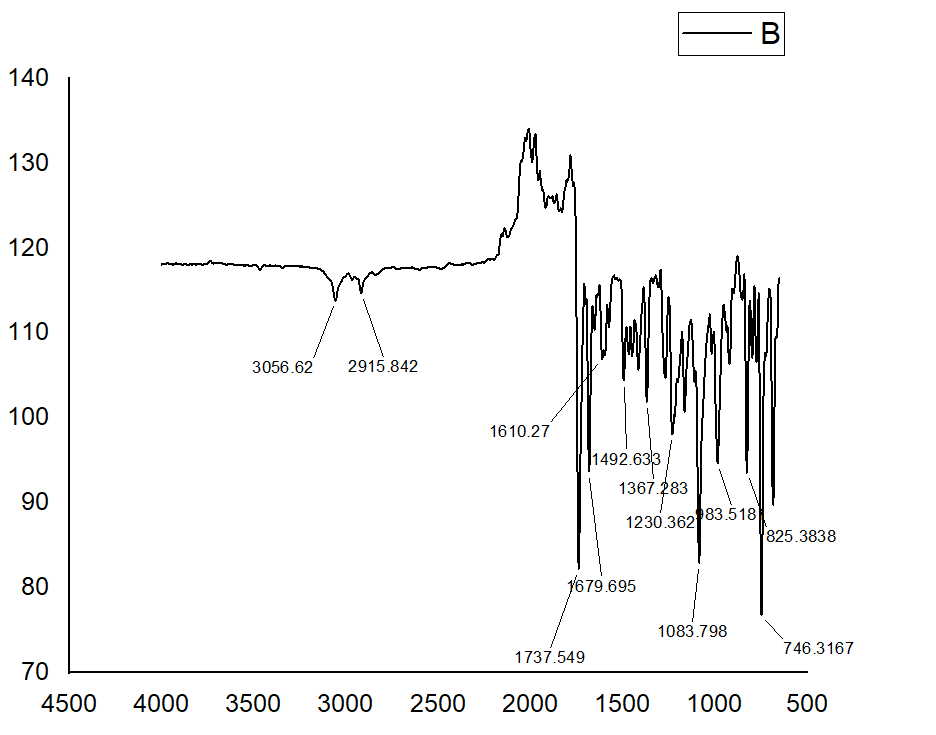

**Figure 9S. Compound 3c ^1^H NMR**


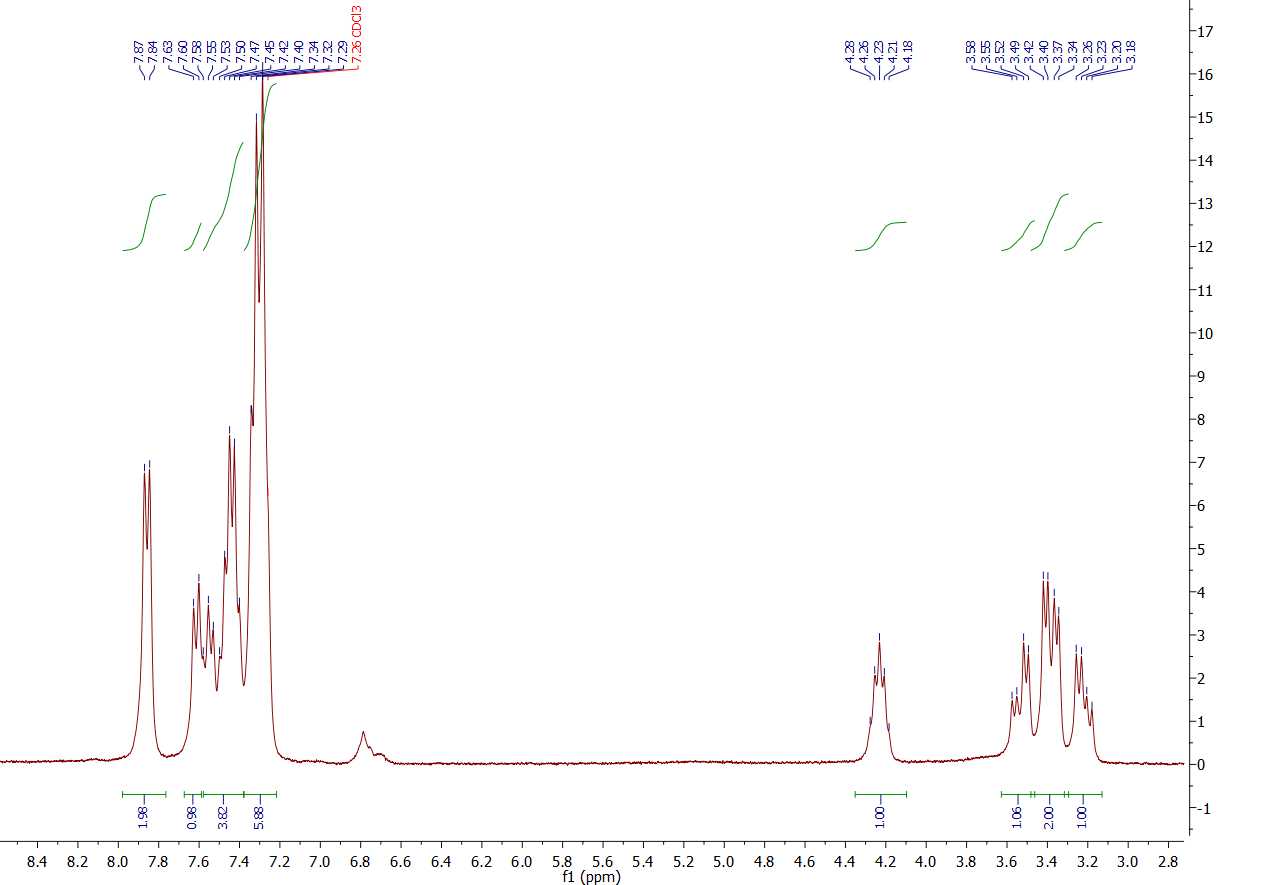

**Figure 10S. Compound 3c ^13^C NMR**

**
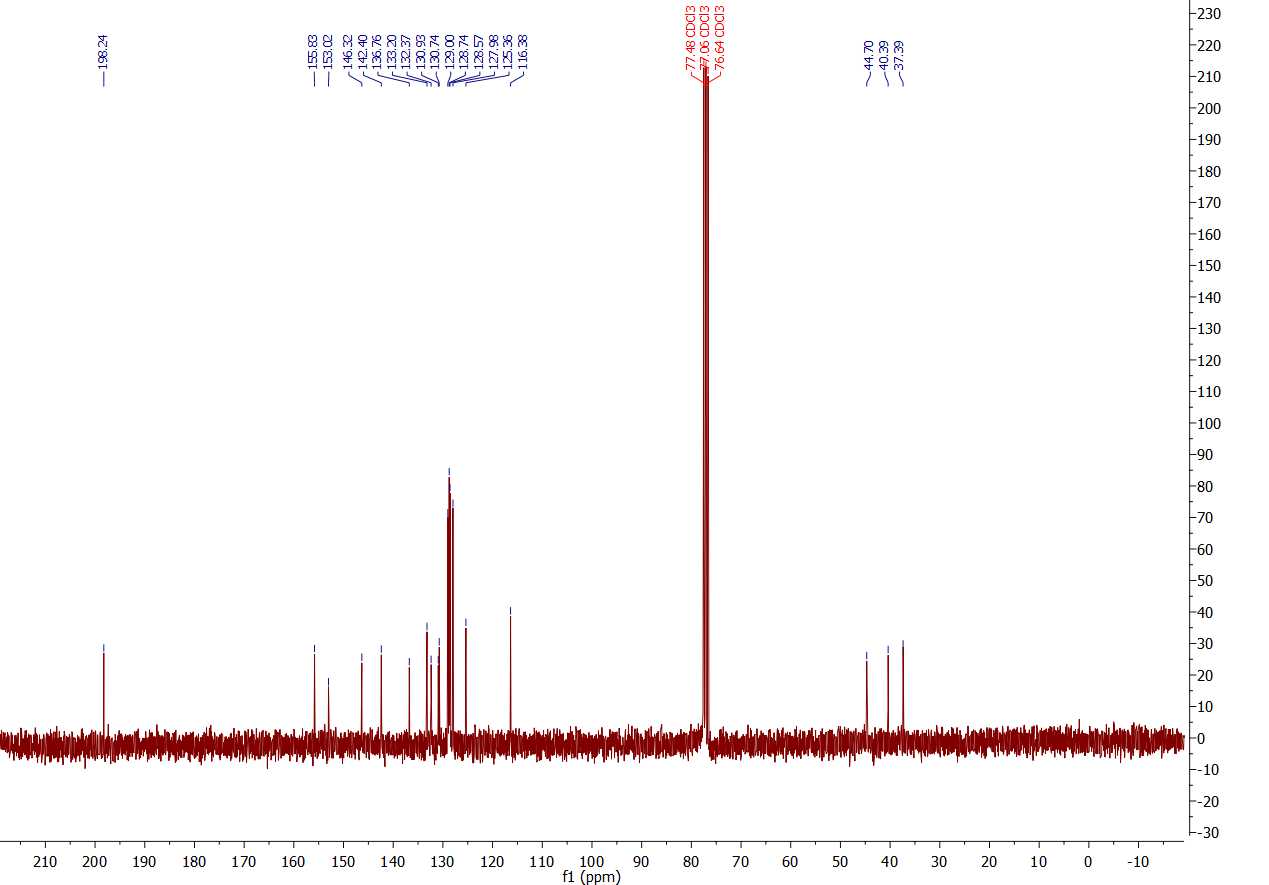
**

**Figure 11S. Compound 3c MS (m/z) 403 [M^+^]**


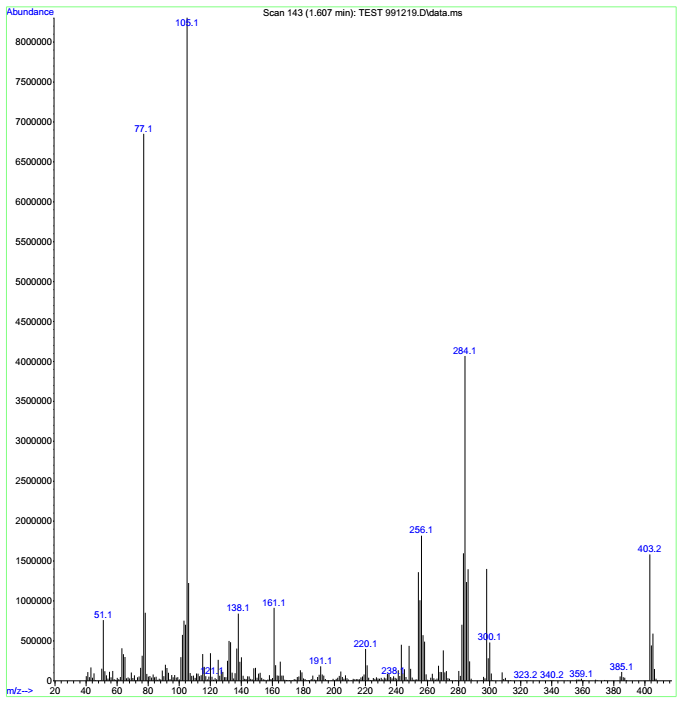

**Figure 12S. Compound 3c** **FT-IR**

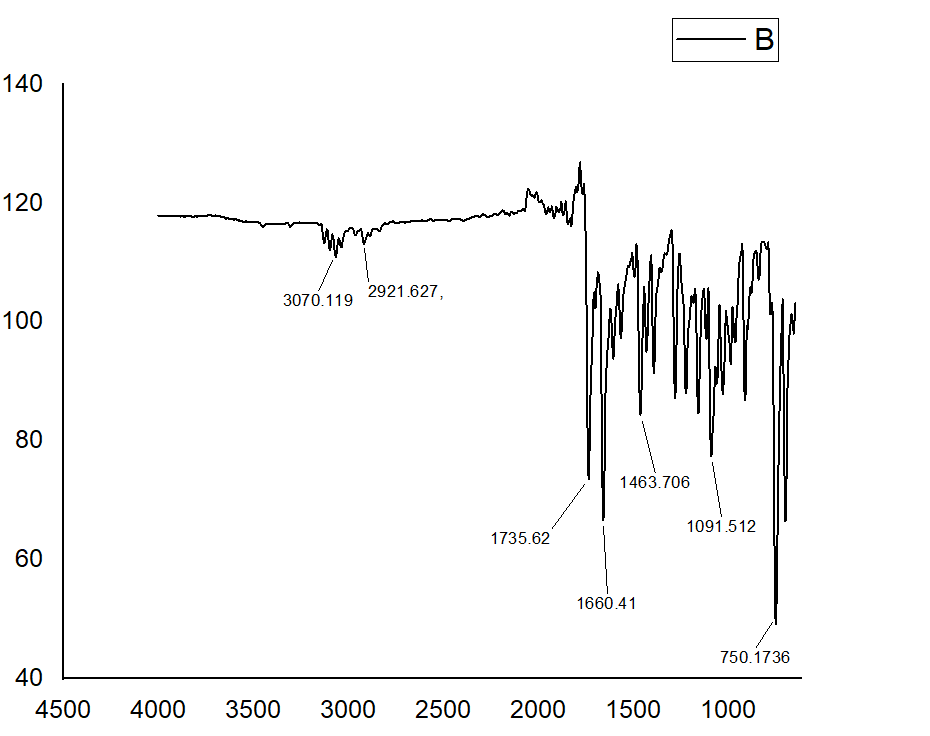


**Figure 13S. Compound 3d ^1^H NMR**

**
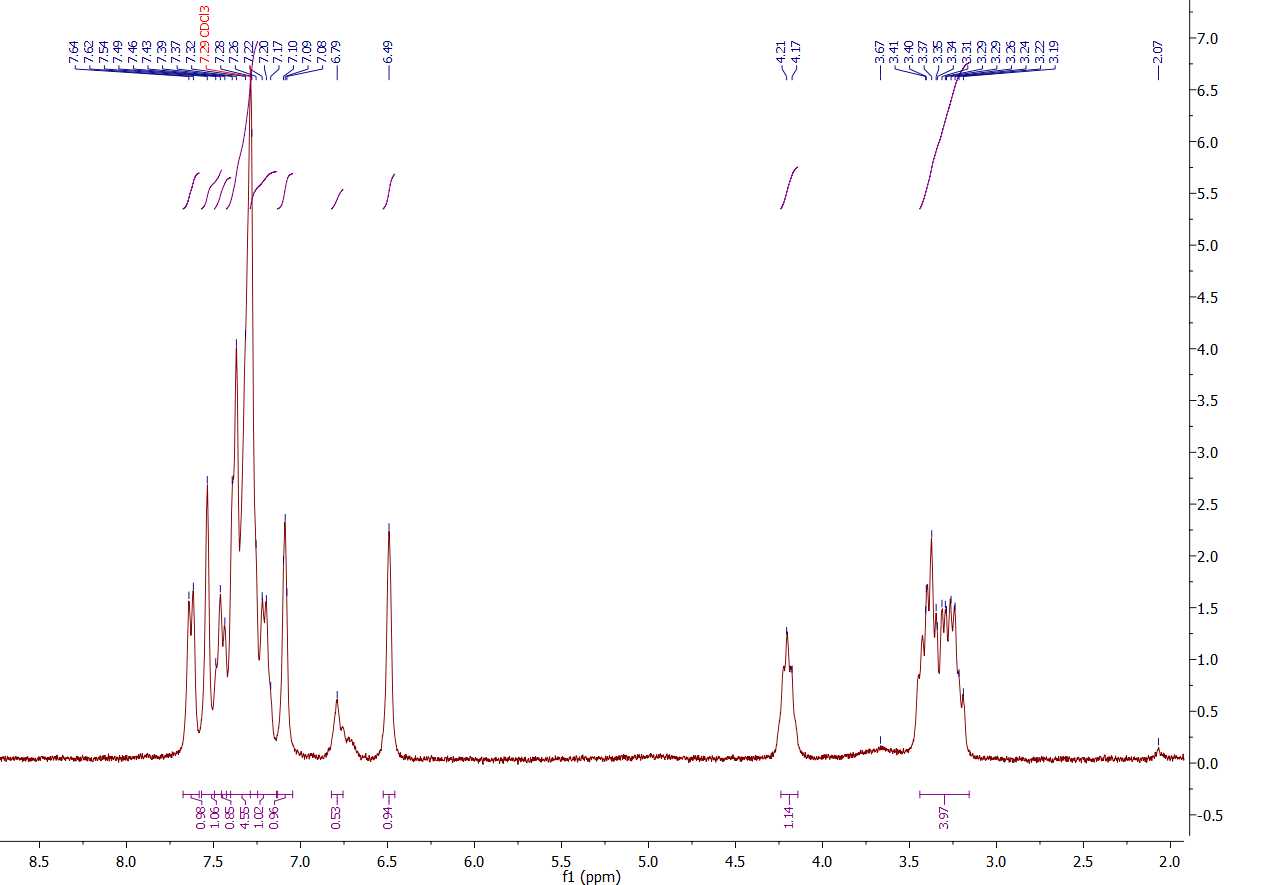
**

**Figure 14S. Compound 3d ^13^C NMR**

**
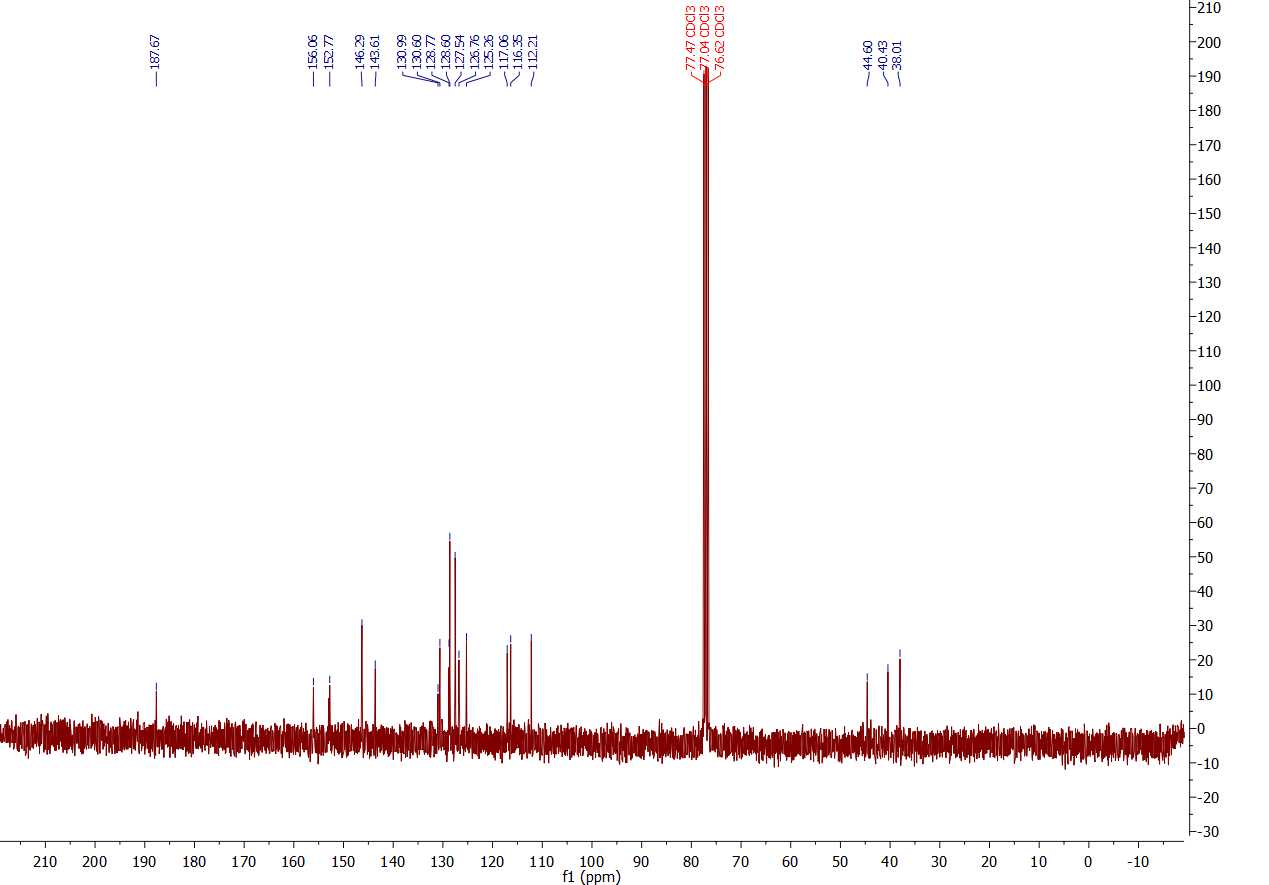
**

**Figure 15S. Compound 3d MS (m/z) 359 [M^+^]**


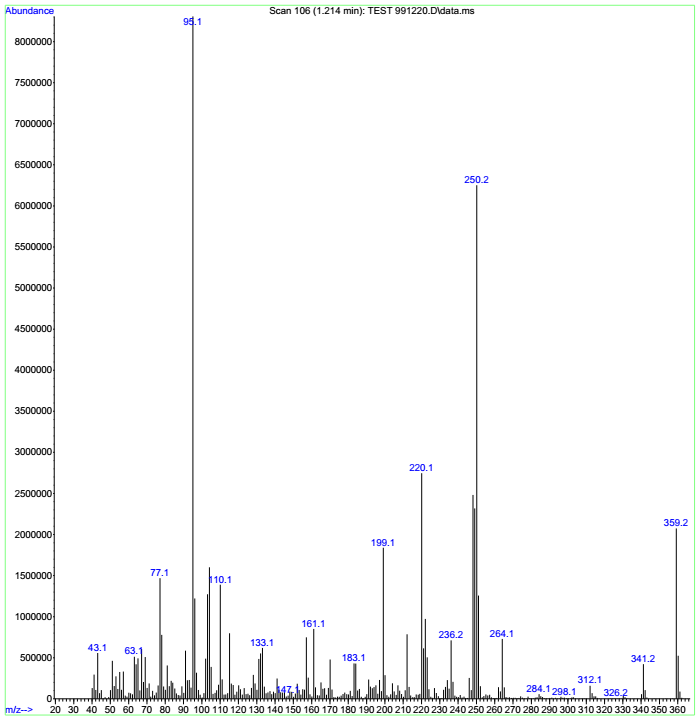

**Figure 16S. Compound 3e ^1^H NMR**

**
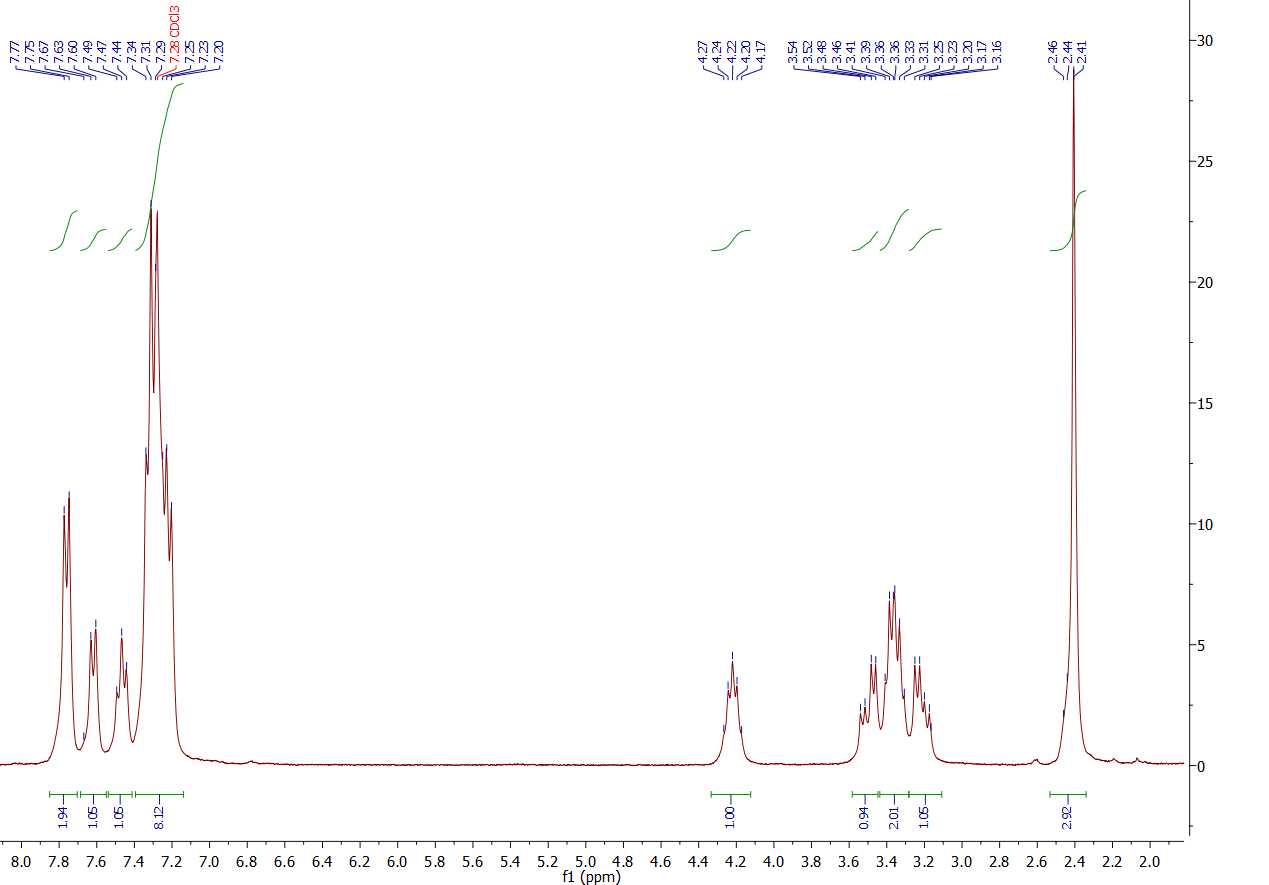
**

**Figure 17S. Compound 3e ^13^C NMR**

**
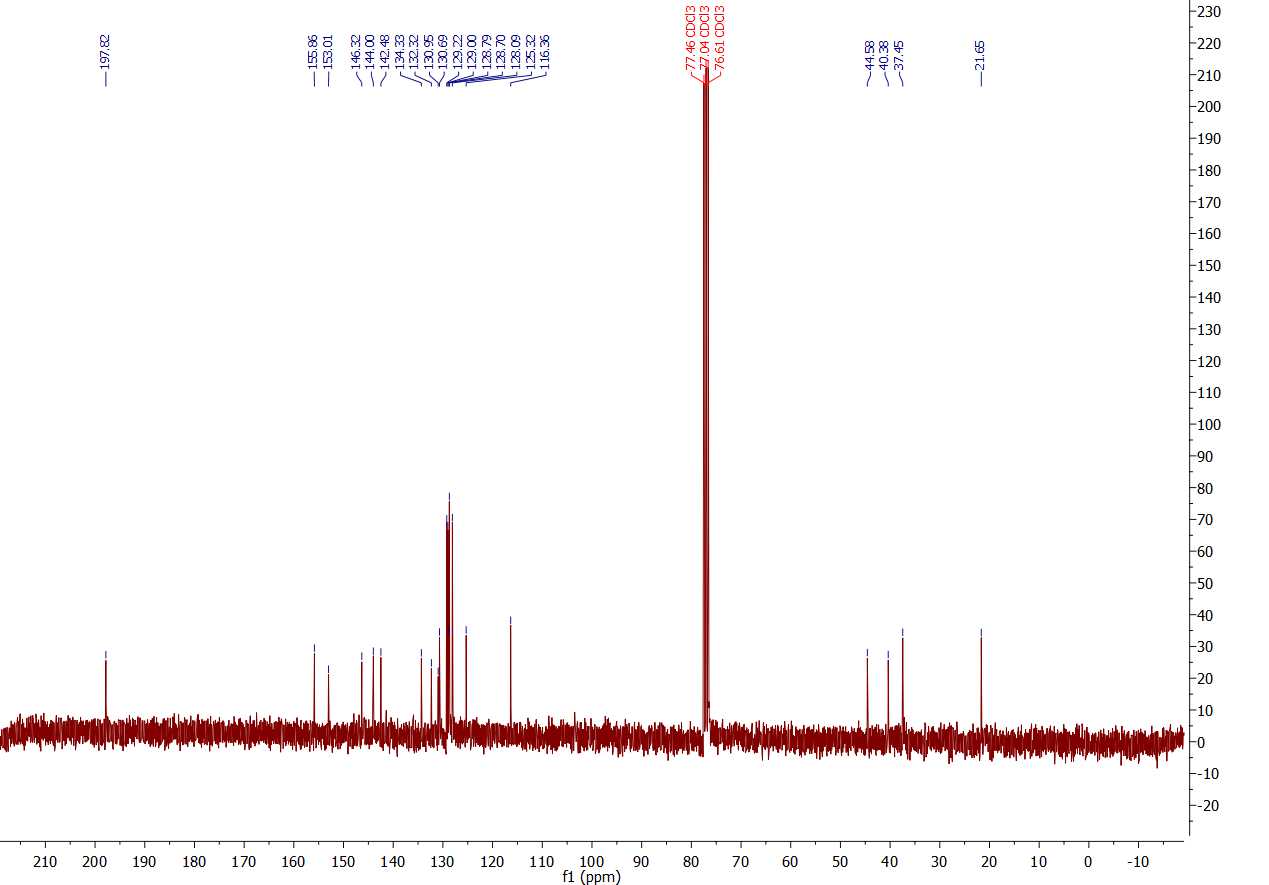
**

**Figure 18S. Compound 3e MS (m/z) 417 [M^+^]**


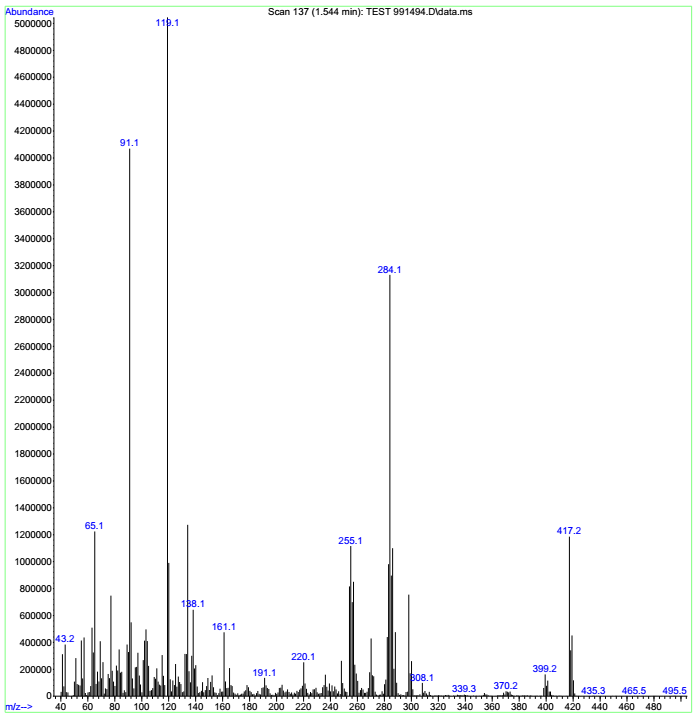

**Figure 19S. Compound 3f ^1^H NMR**

**
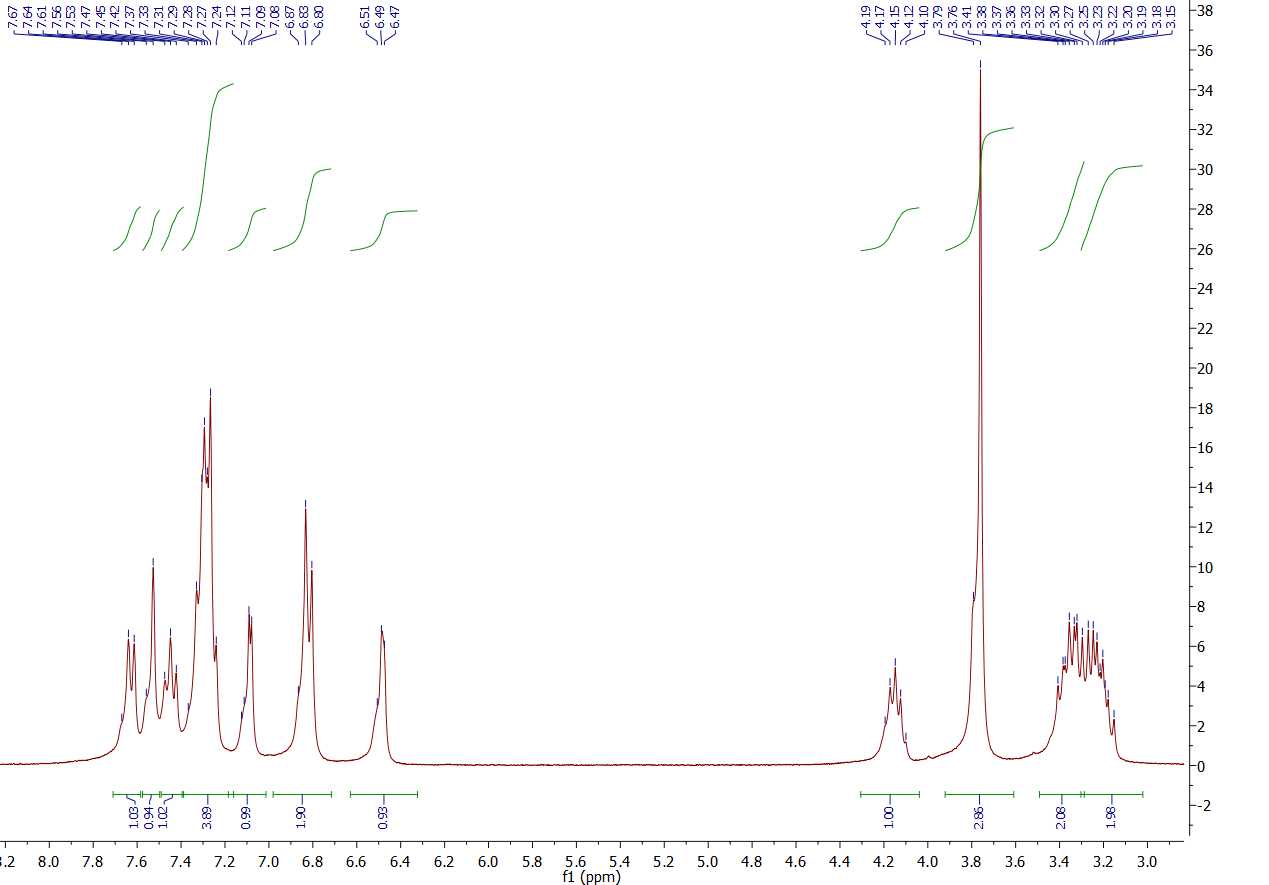
**

**Figure 20S. Compound 3f ^13^C NMR**

**
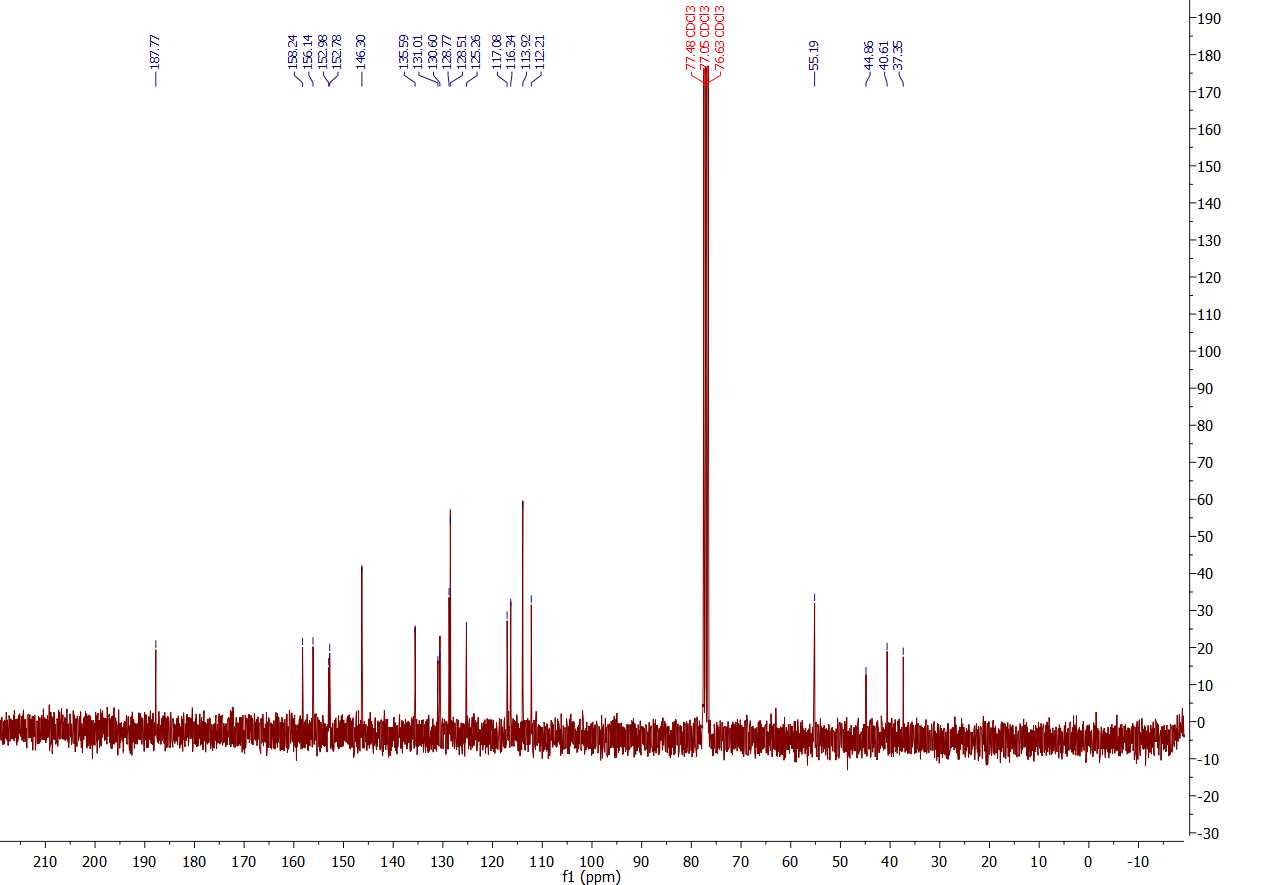
**

**Figure 21S.Compound 3f MS (m/z) 389 [M^+^]**


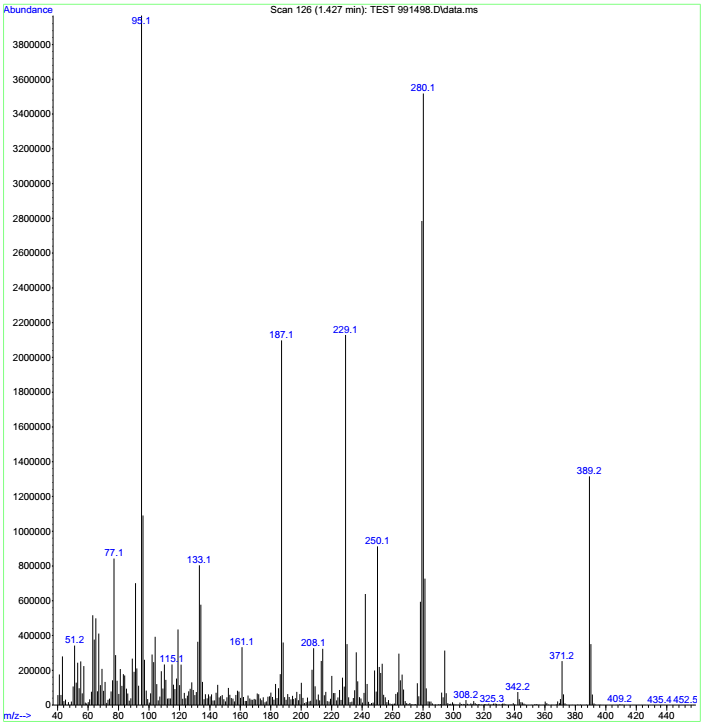

**Figure 22S. Compound 3g ^1^H NMR**

**
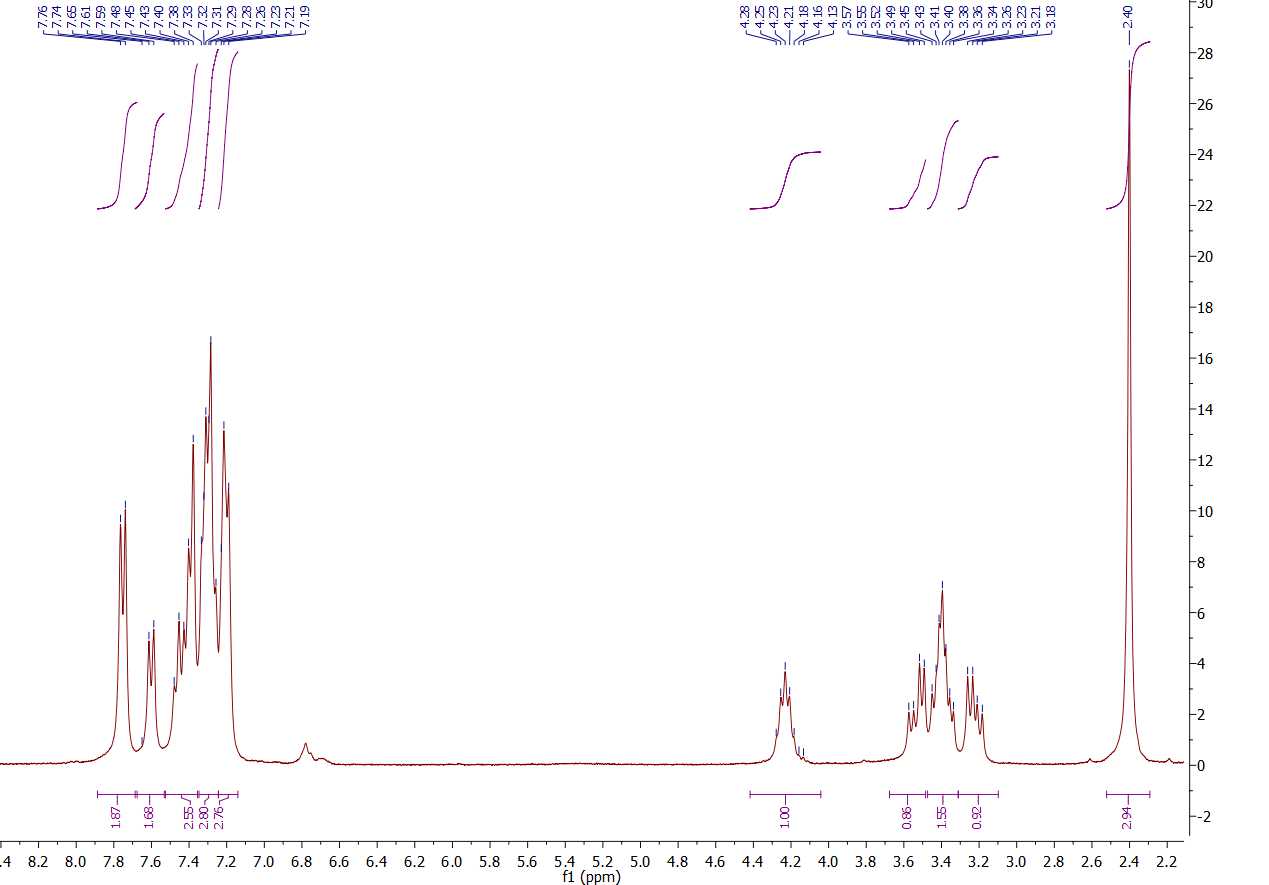
**

**Figure 23S. Compound 3g ^13^C NMR**

**
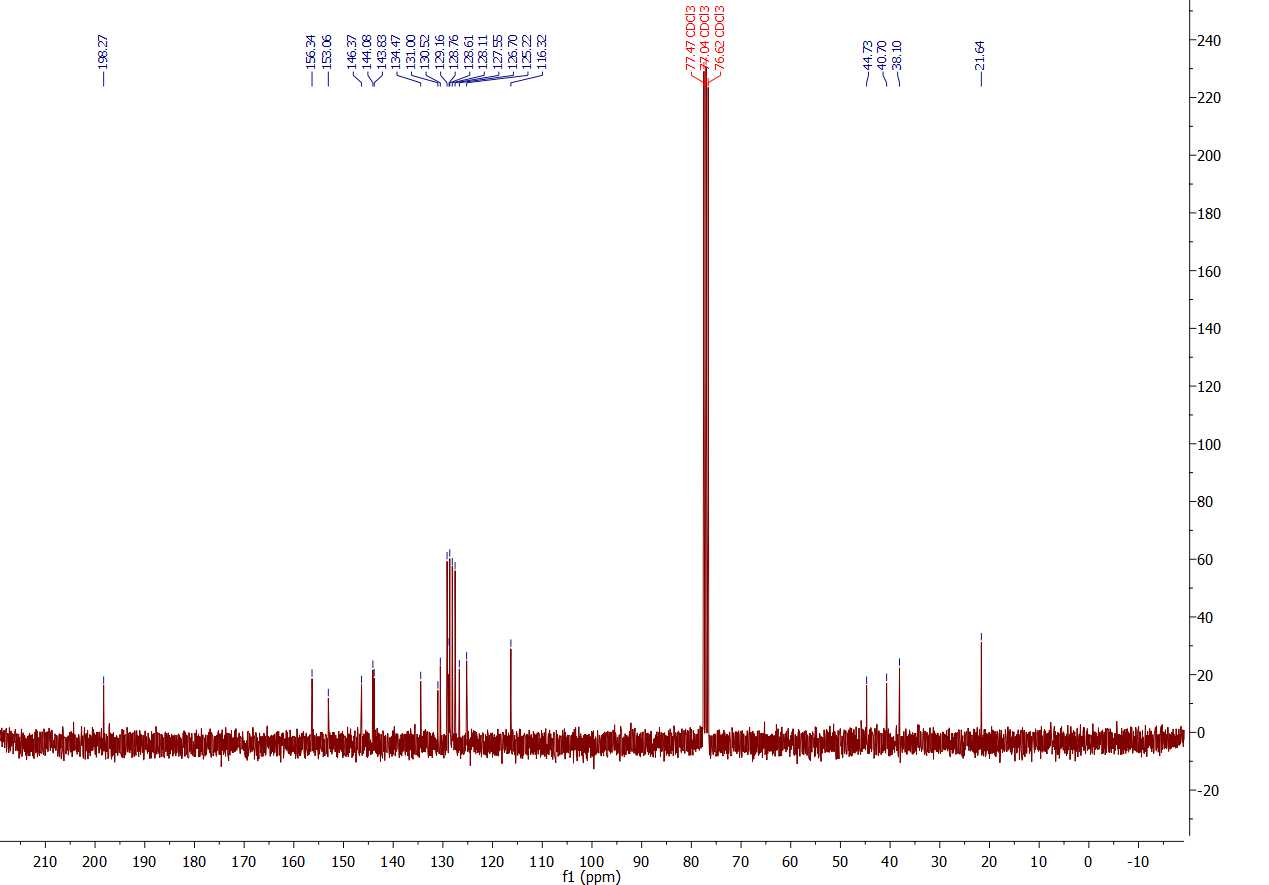
**

**Figure 24S. Compound 3g MS (m/z) 383 [M^+^]**


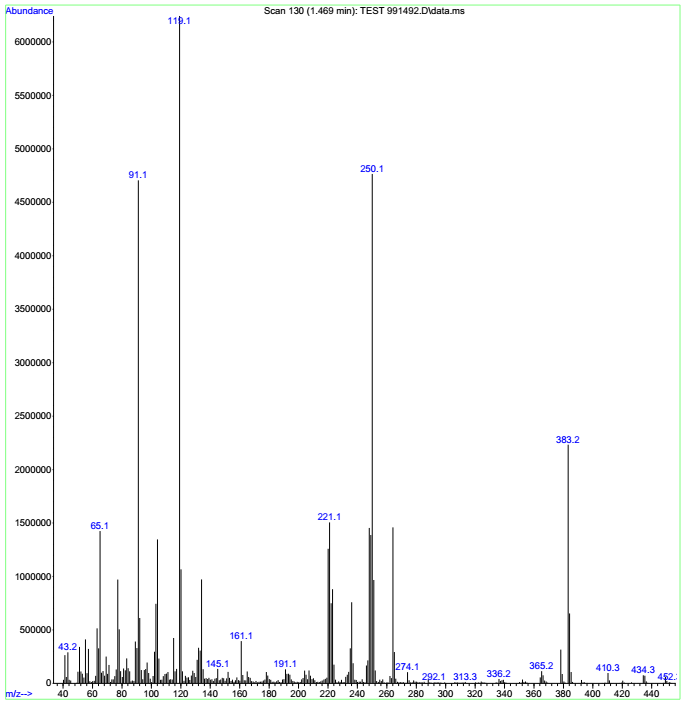

**Figure 25S. Compound 3h ^1^H NMR**

**
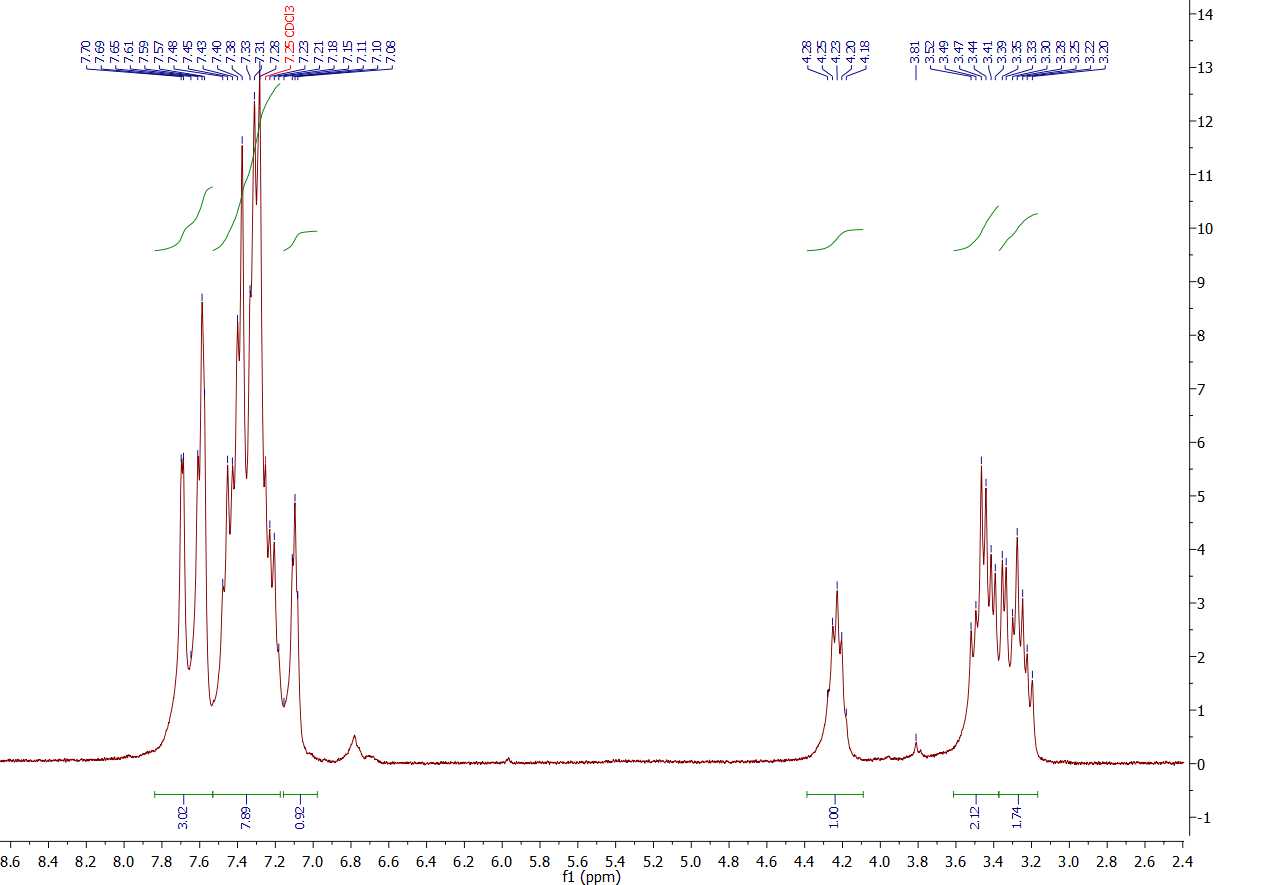
**

**Figure 26S. Compound 3h ^13^C NMR**

**
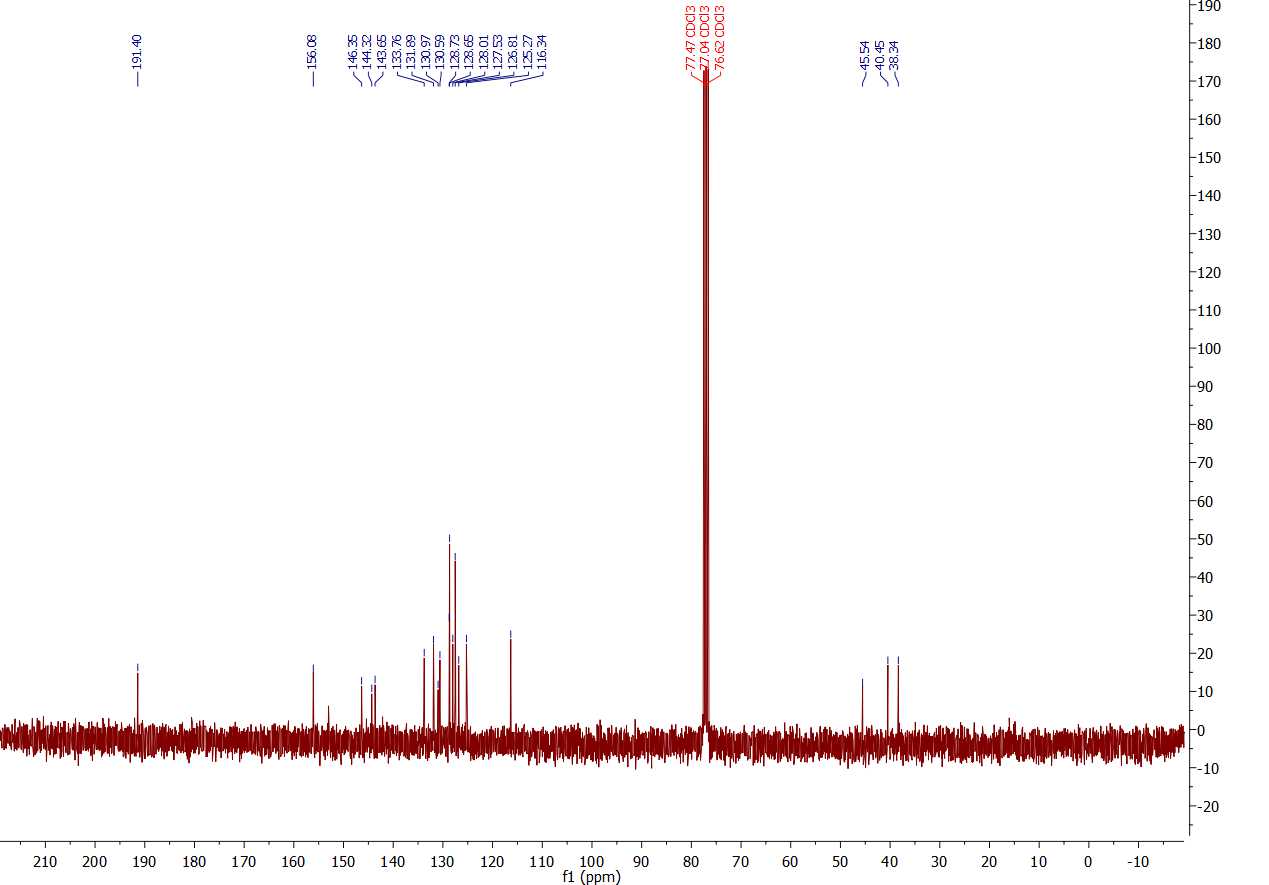
**

**Figure 27S. Compound 3h MS (m/z) 375 [M^+^]**


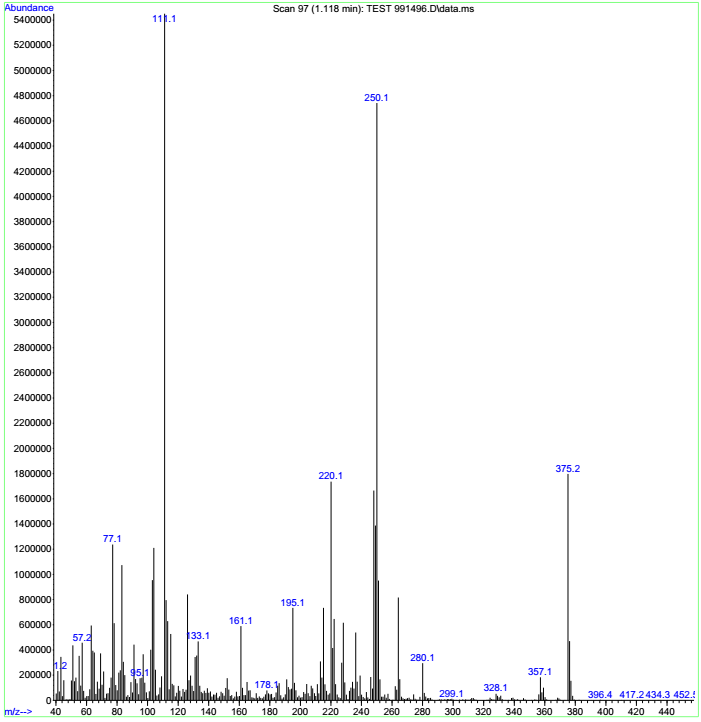

**Figure 28S. Compound 3i ^1^H NMR**

**
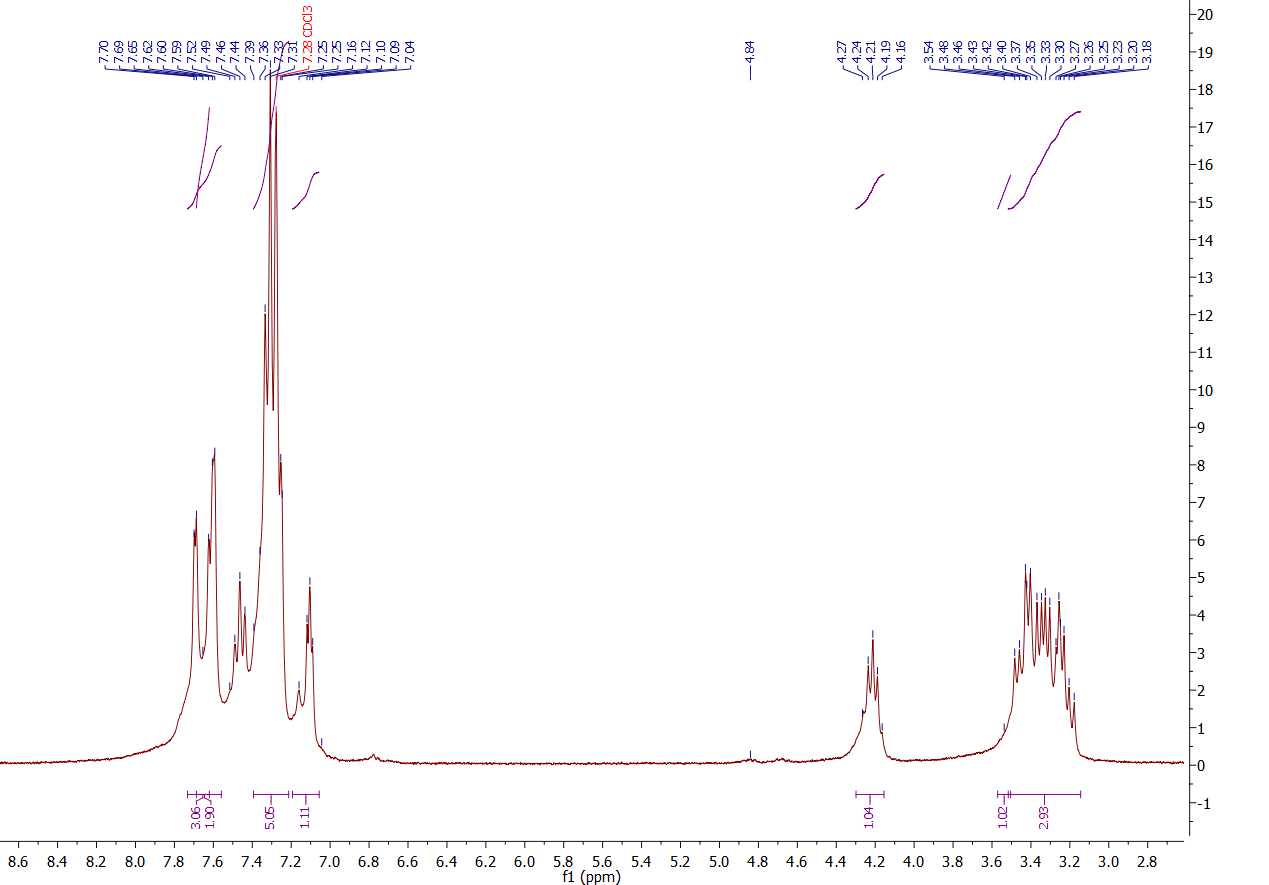
**

**Figure 29S. Compound 3i ^13^C NMR**

**
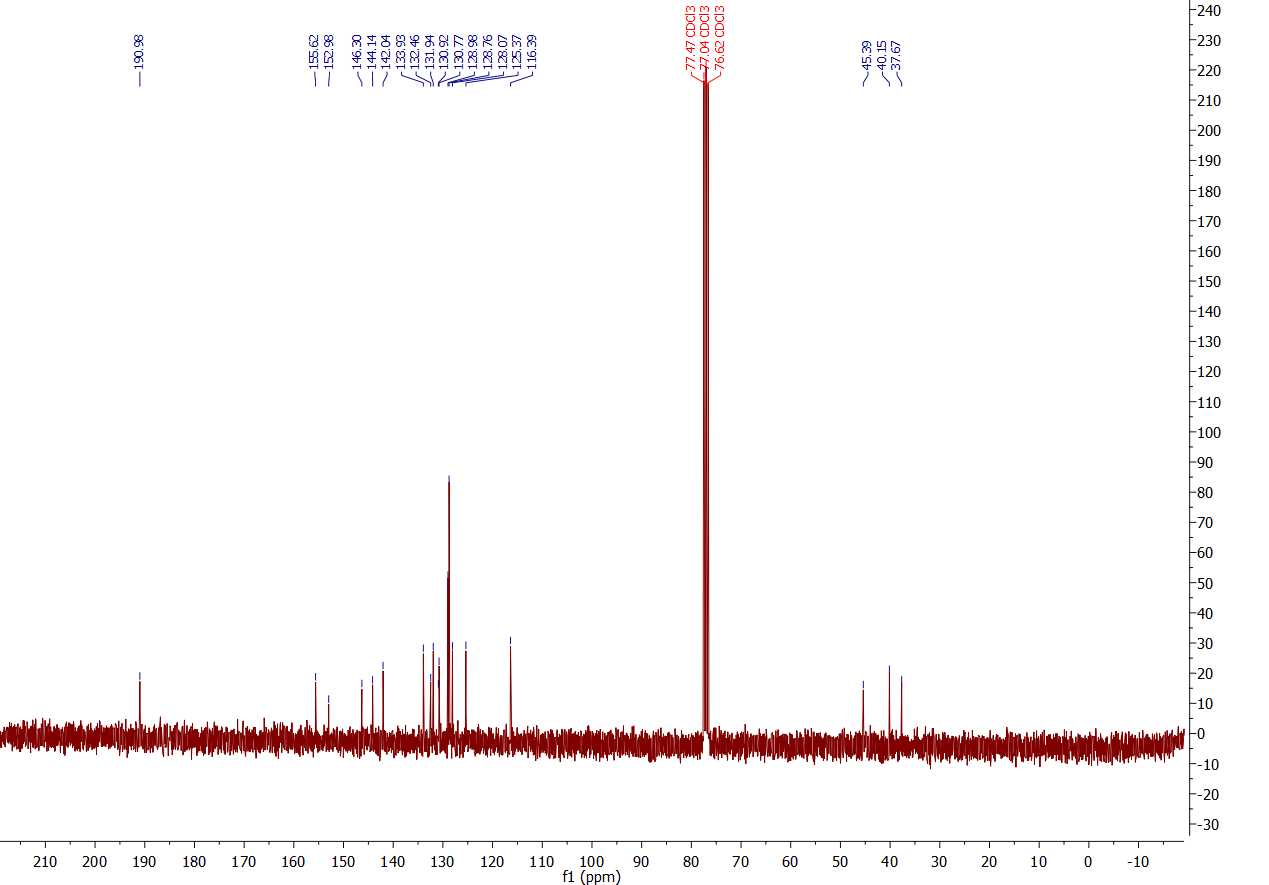
**

**Figure 30S. Compound 3i MS (m/z) 409 [M^+^]**


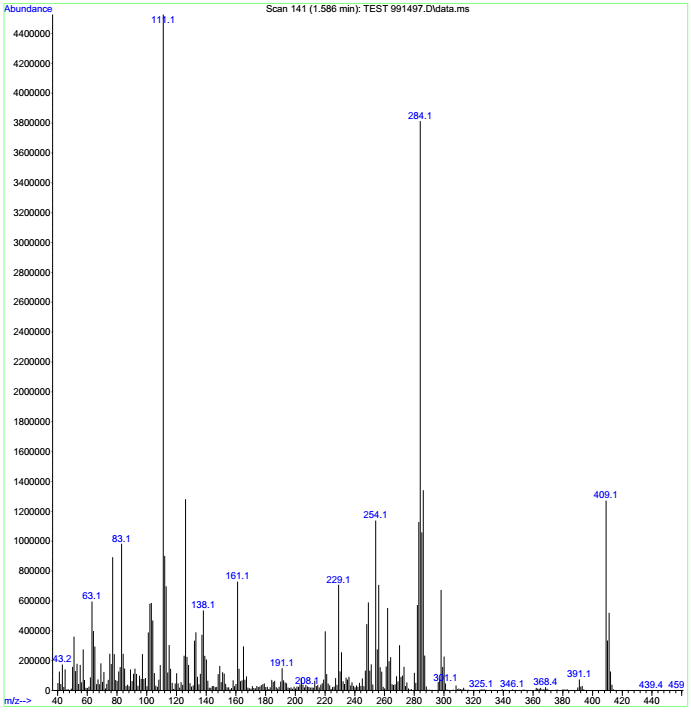

**Figure 31S. Compound 3j ^1^H NMR**


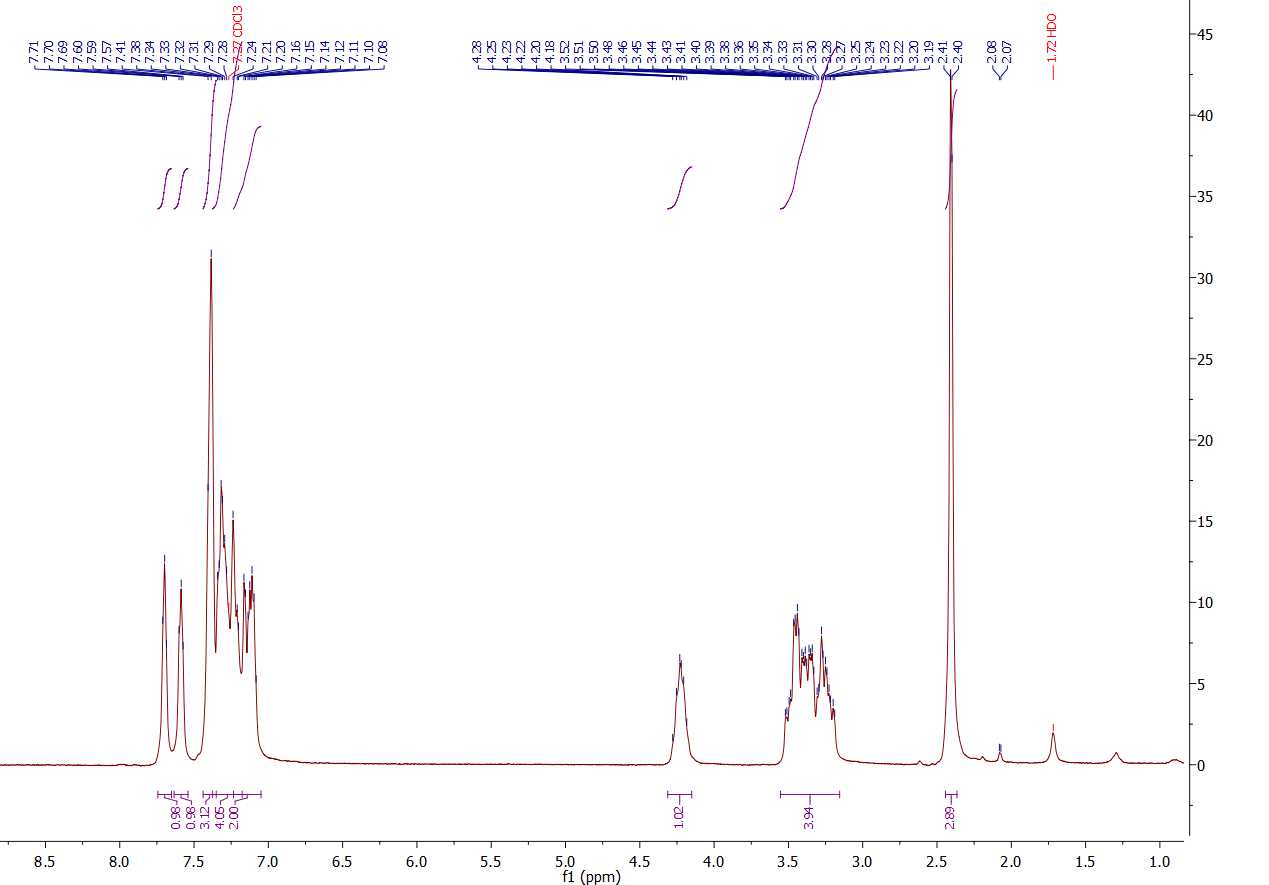

**Figure 32S. Compound 3j ^13^C NMR**

**
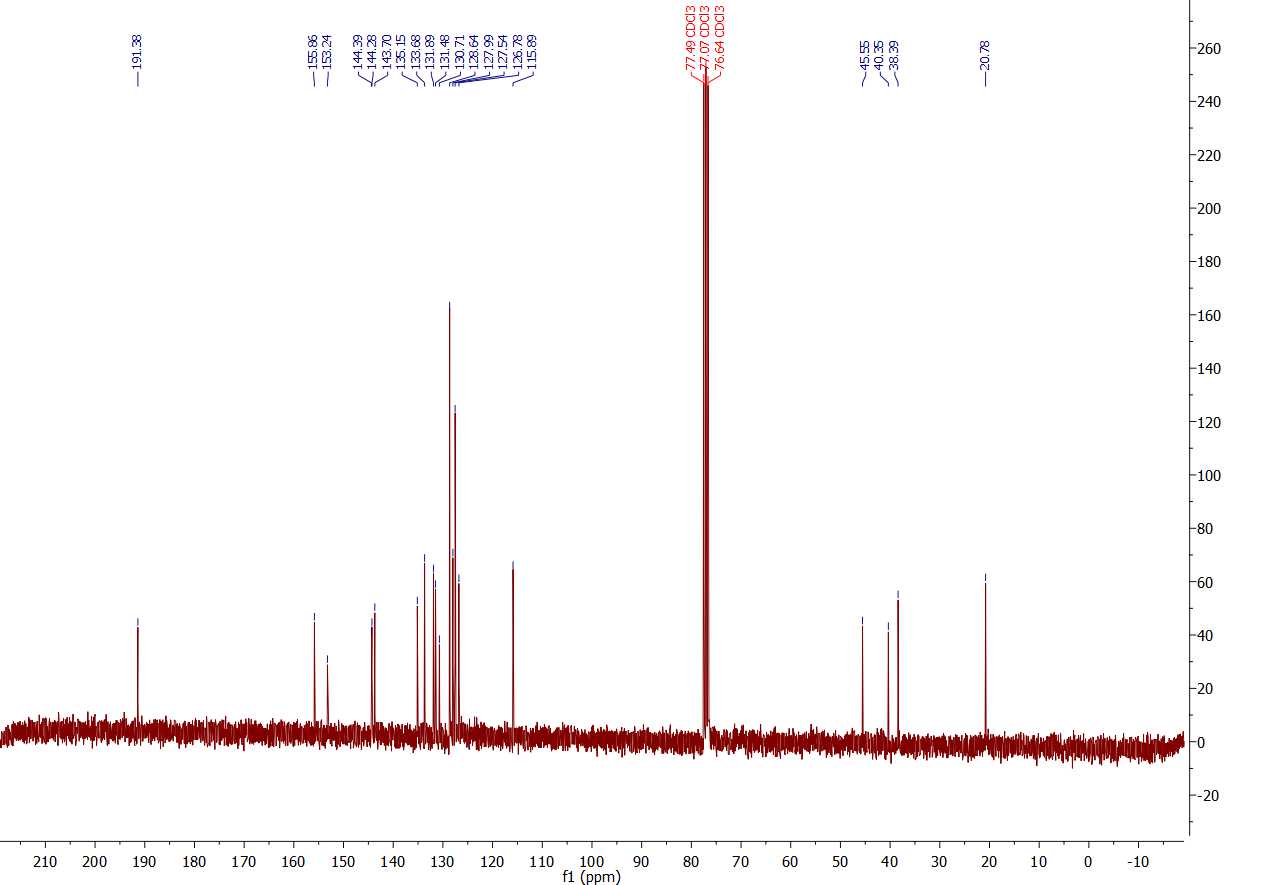
**

**Figure 33S. Compound 3j MS (m/z) 389 [M^+^]**


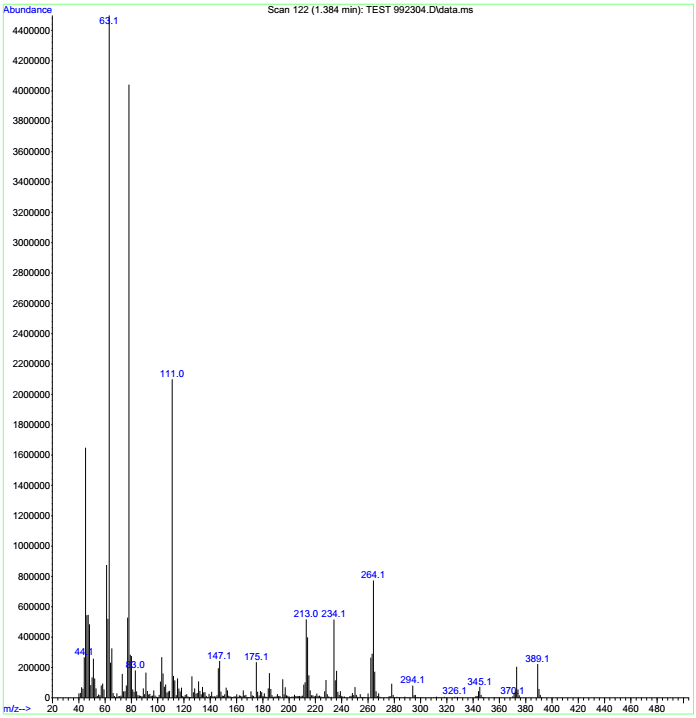

**Figure 34S. Compound 3k ^1^H NMR**

**
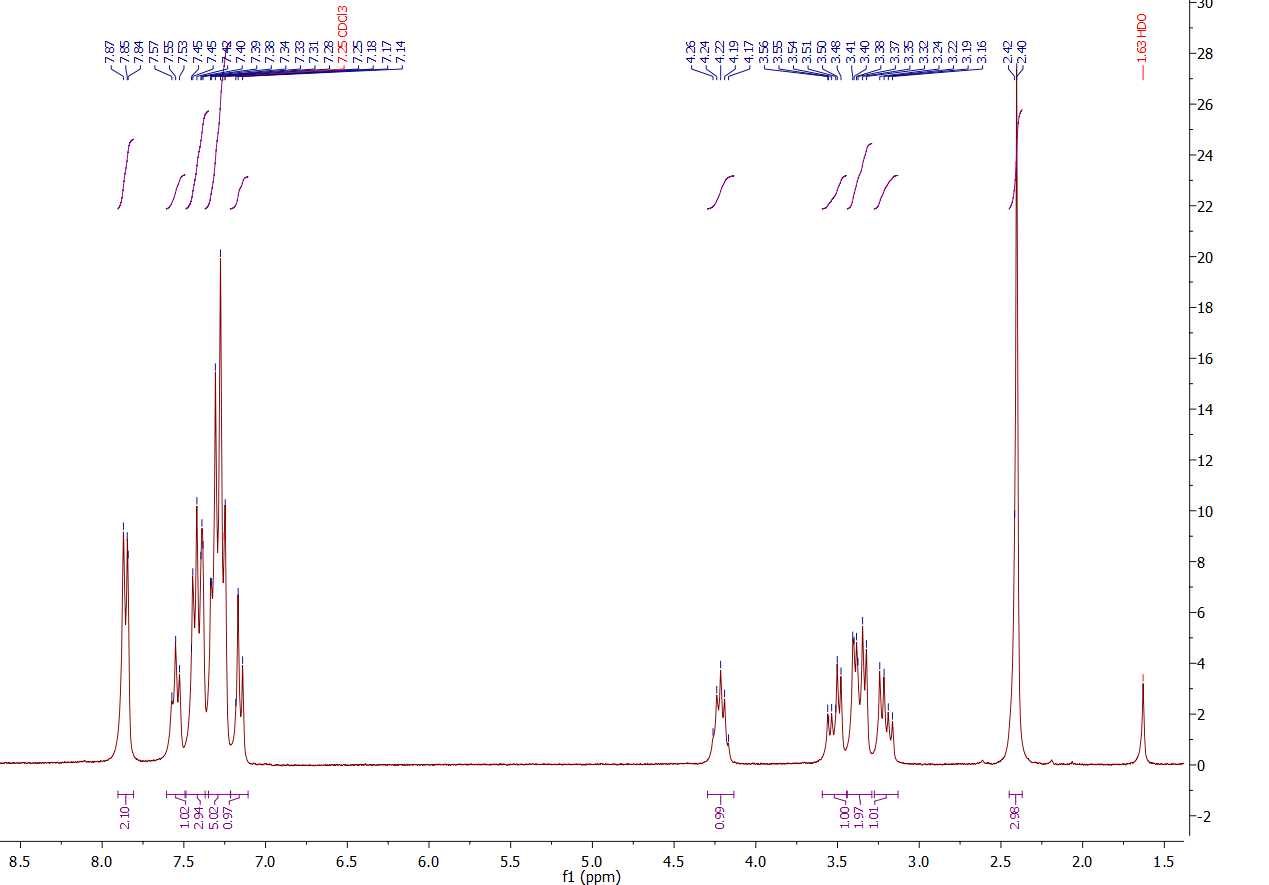
**

**Figure 35S. Compound 3k ^13^C NMR**

**
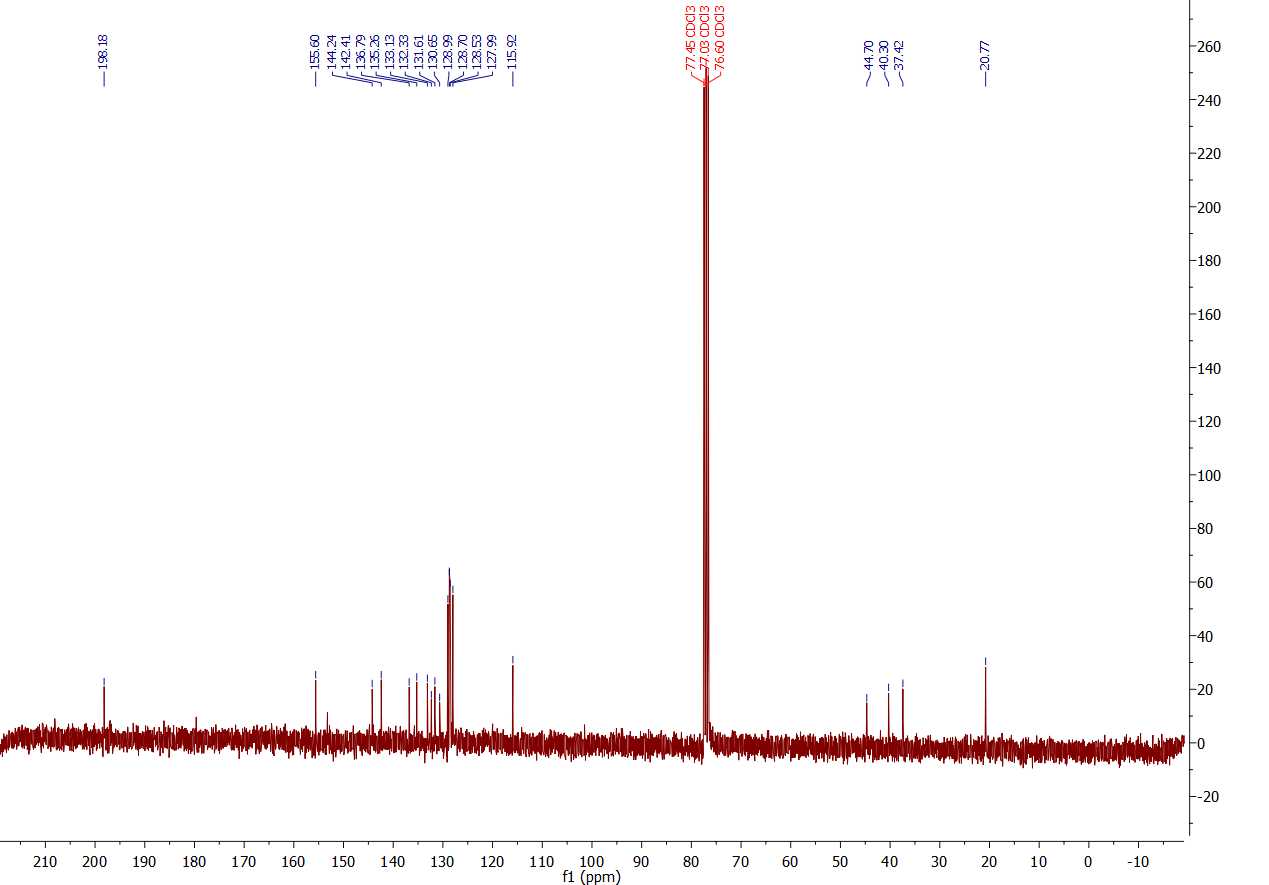
**

**Figure 36S. Compound 3k MS (m/z) 417 [M^+^]**


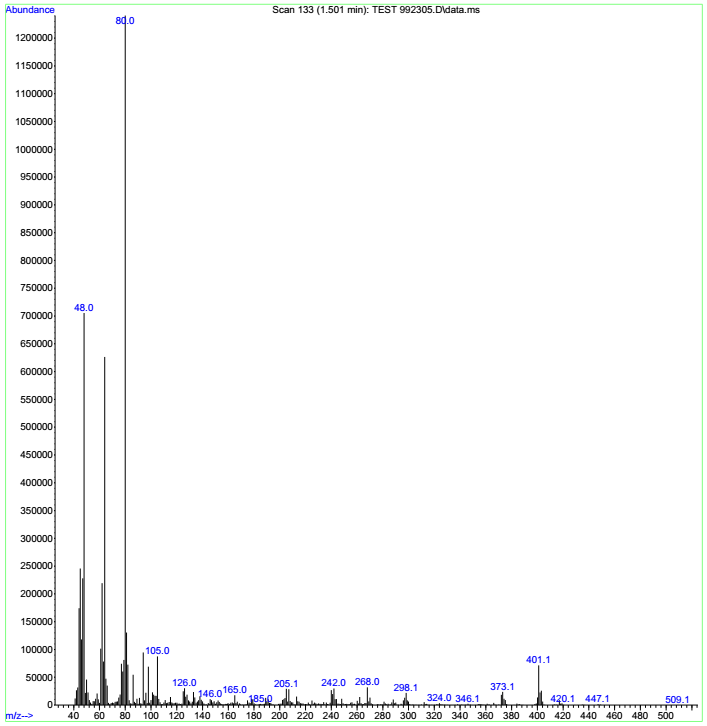

**Figure 37S. Compound 3l ^1^H NMR**


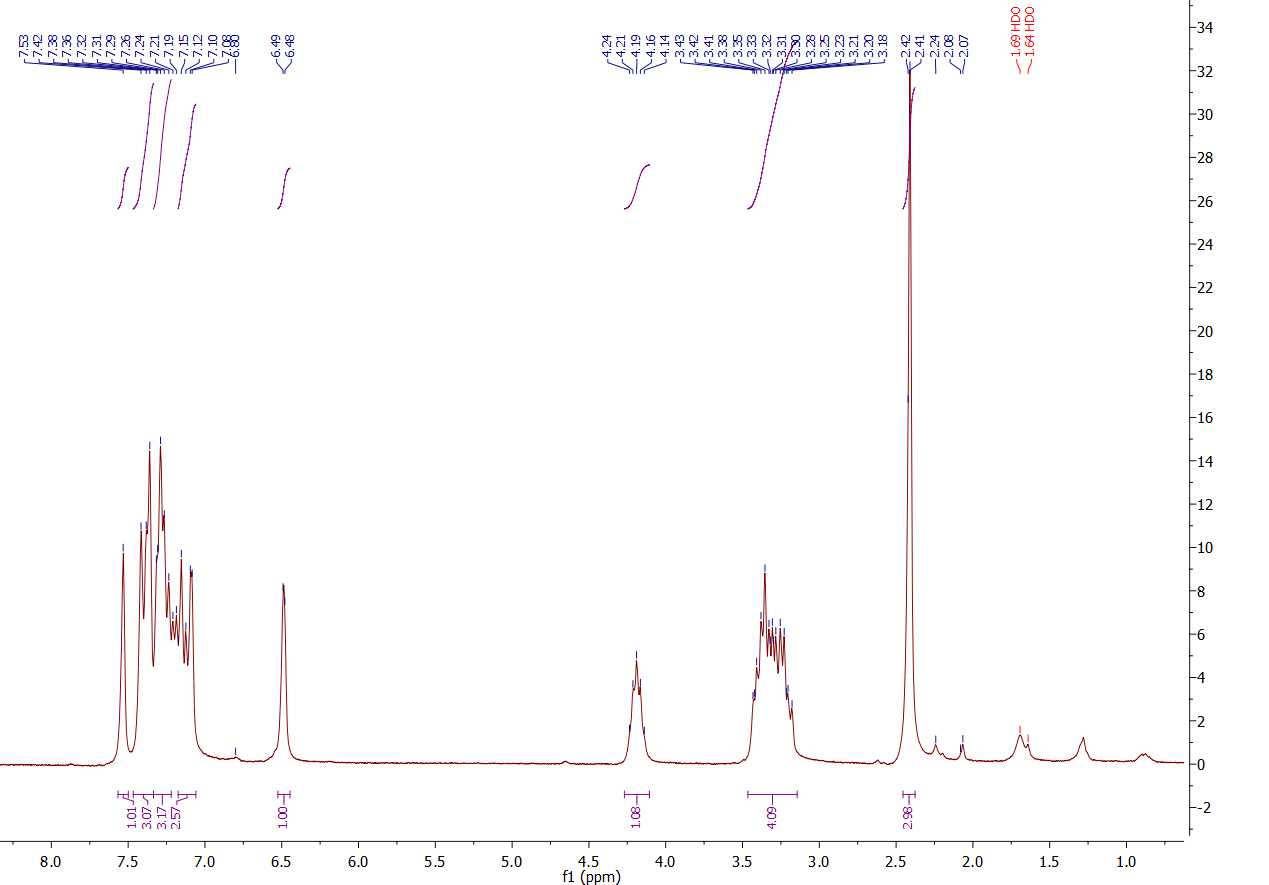

**Figure 38S. Compound 3l ^13^C NMR**

**
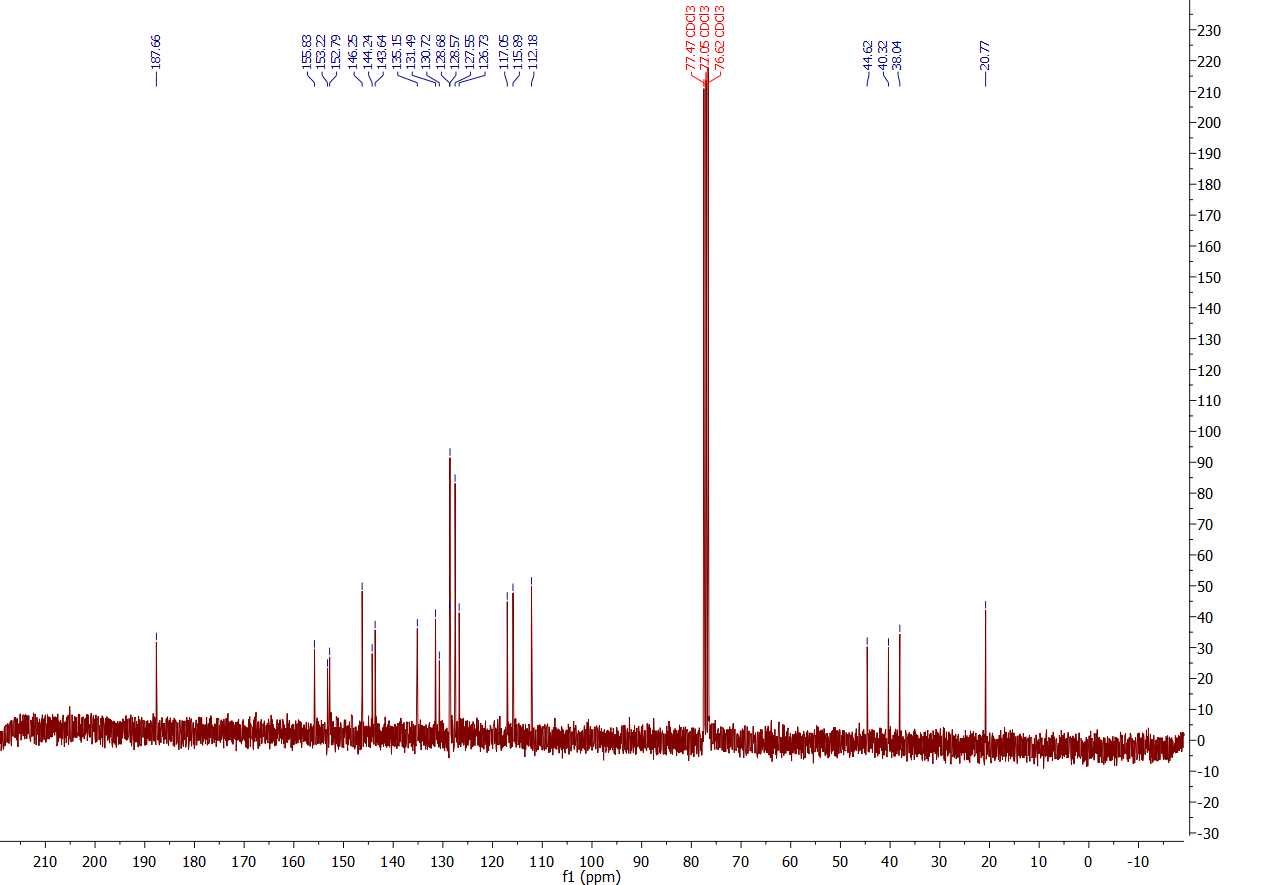
**

**Figure 39S. Compound 3l MS (m/z) 373 [M^+^]**


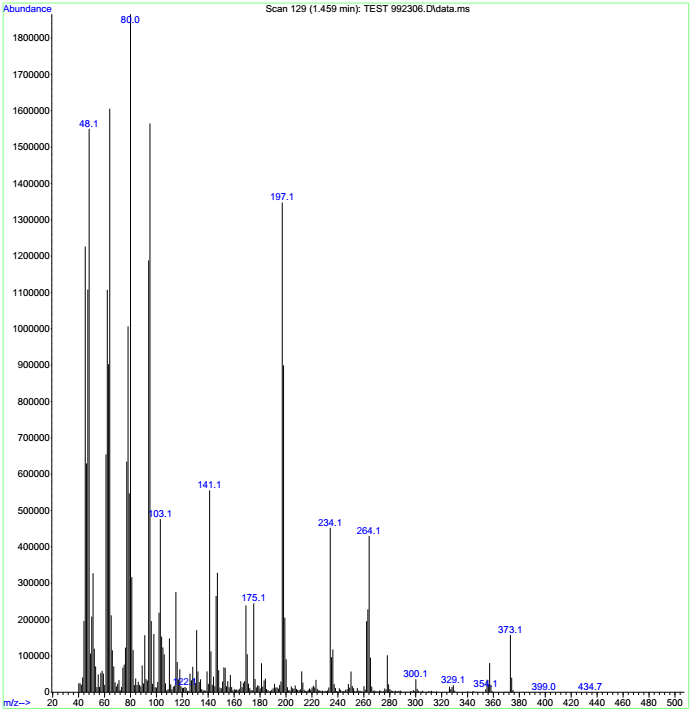

**Figure 40S. Compound 3m ^1^H NMR**

**
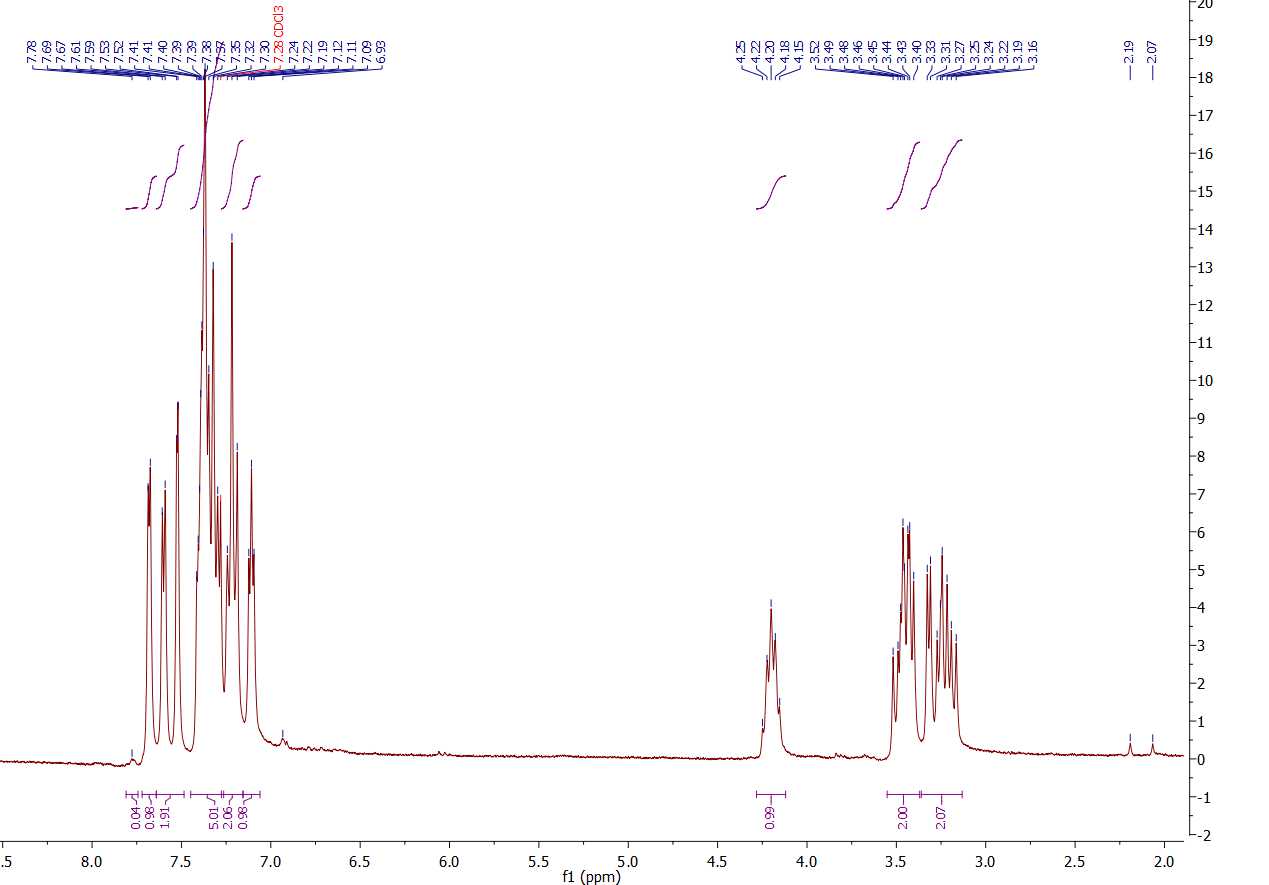
**

**Figure 41S. Compound 3m ^13^C NMR**

**
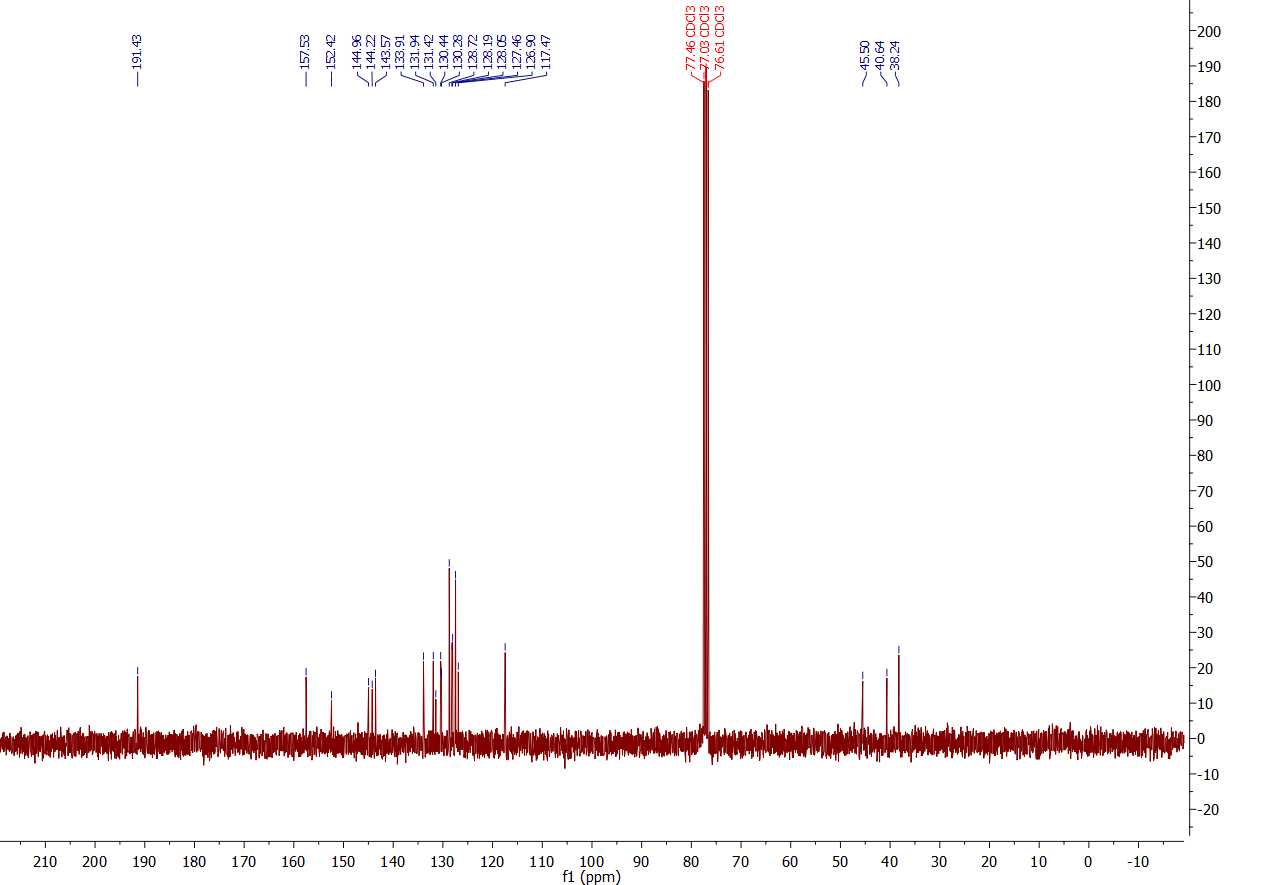
**

**Figure 42S. Compound 3m MS (m/z) 409 [M^+^]**


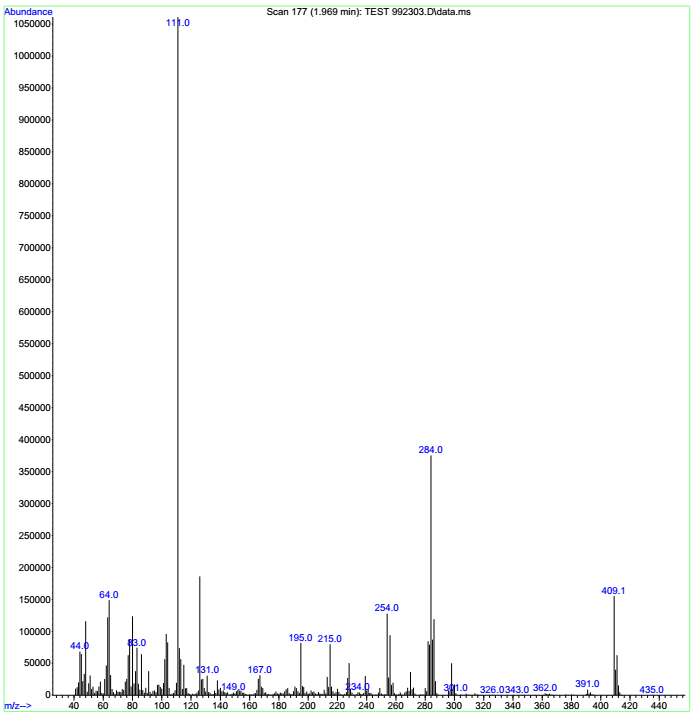

Supplement: Supplementary file 1 — Supplementary Information. [file 41598_2022_16291_MOESM1_ESM.docx]
